# Association Mapping for 24 Traits Related to Protein Content, Gluten Strength, Color, Cooking, and Milling Quality Using Balanced and Unbalanced Data in Durum Wheat [*Triticum turgidum* L. var. *durum* (Desf).]

**DOI:** 10.3389/fgene.2019.00717

**Published:** 2019-08-16

**Authors:** Marina Johnson, Ajay Kumar, Atena Oladzad-Abbasabadi, Evan Salsman, Meriem Aoun, Frank A. Manthey, Elias M. Elias

**Affiliations:** Department of Plant Sciences, North Dakota State University, Fargo, ND, United States

**Keywords:** durum wheat, genome wide association mapping, gluten strength, grain and pasta color, Infinium iSelect 90k, pasta, protein content, unbalanced data

## Abstract

Durum wheat [*Triticum durum* (Desf).] is mostly used to produce pasta, couscous, and bulgur. The quality of the grain and end-use products determine its market value. However, quality tests are highly resource intensive and almost impossible to conduct in the early generations in the breeding program. Modern genomics-based tools provide an excellent opportunity to genetically dissect complex quality traits to expedite cultivar development using molecular breeding approaches. This study used a panel of 243 cultivars and advanced breeding lines developed during the last 20 years to identify SNPs associated with 24 traits related to nutritional value and quality. Genome-wide association study (GWAS) identified a total of 179 marker–trait associations (MTAs), located in 95 genomic regions belonging to all 14 durum wheat chromosomes. Major and stable QTLs were identified for gluten strength on chromosomes 1A and 1B, and for PPO activity on chromosomes 1A, 2B, 3A, and 3B. As a large amount of unbalance phenotypic data are generated every year on advanced lines in all the breeding programs, the applicability of such a dataset for identification of MTAs remains unclear. We observed that ∼84% of the MTAs identified using a historic unbalanced dataset (belonging to a total of 80 environments collected over a period of 16 years) were also identified in a balanced dataset. This suggests the suitability of historic unbalanced phenotypic data to identify beneficial MTAs to facilitate local-knowledge-based breeding. In addition to providing extensive knowledge about the genetics of quality traits, association mapping identified several candidate markers to assist durum wheat quality improvement through molecular breeding. The molecular markers associated with important traits could be extremely useful in the development of improved quality durum wheat cultivars using marker-assisted selection (MAS).

## Introduction

Durum wheat, the hardest of all wheats (Miller et al. 1982), is one of the most important crops in the world. Annual worldwide durum wheat production is estimated to be around 36 million tons, with approximately, 2.5 million tons produced in the United States (NASS, 2018). The market price of durum wheat is generally higher than other wheat classes, which makes durum wheat attractive for growers. Apart from grain yield and disease resistance, quality is a strong criterion of durum wheat variety selection. Grain quality parameters differ based on the end use but usually include visual appearance, vitreousness (VIT), weather damage (falling number), and protein quantity and quality. During milling, durum wheat endosperm is ground into coarse particles called semolina, which is then used for making pasta and couscous. The quality of semolina is determined by many factors including ash content, color, and particle-size distribution. Grain protein concentration is important for high-quality pasta production due to its effect on the firmness of cooked pasta and tolerance to overcooking (Dexter and Matsuo, 1977). Gluten strength can also affect pasta characteristics such as tolerance to overcooking, reduced stickiness, and minimal loss of solids during cooking. The degree of kernel translucency, and thus the apparent degree of vitreousness, is related to the degree of kernel compactness. Generally, more desirable coarse semolina is produced from highly vitreous wheats (Hoseney 1987). Milling yield is another important criterion for the milling industry, as higher semolina yield means higher profits for durum wheat millers. Pasta color is an important consideration by the consumers. Therefore, processors prefer clear, bright yellow semolina, which generally produces a superior end product. Polyphenol oxidases (PPOs) produce dark/brown polyphenols (Anderson and Morris, 2003). When PPO activity reaches substantial levels, an unappealing brown color can occur in end products (Sissons, 2008). Cooked quality for pasta, which is determined by its cooked firmness, cooking loss, and cooked weight, is important to producers. Durum wheat with high grain and end-product quality can receive premium prices in national and international markets.

The phenotypic evaluation of many important quality traits requires large amounts of grain per line, which is not feasible in early generations of breeding due to the large number of lines and limited resources in most breeding programs. Marker-assisted selection provides an opportunity for breeders to identify superior lines in early generations, thus saving significant resources and speeding up the process of cultivar development. However, most of the quality traits in wheat are quantitatively inherited and influenced by multiple QTL, QTL × QTL, the environment, and the interaction between QTL and environment (McCartney et al., 2005; Li et al., 2012; Kumar et al., 2013; Tadesse et al., 2015; Kumar et al., 2018; Merida-Garcıa et al., 2019). Therefore, a complete knowledge about the genetics of target traits and identification of markers tightly linked to those target traits is essential for the successful integration of MAS in the breeding program. Traits like grain protein content (Blanco et al., 2006; Blanco et al., 2012; Kumar et al., 2018), gluten strength (Kumar et al., 2013; Kumar et al., 2018), and color (Pozniak et al., 2007; Zhang and Dubcovsky, 2008b; Roncallo et al., 2012) have been investigated in some details in durum wheat. However, a limited number of studies have been conducted to genetically dissect other important quality traits related to milling, PPO (Si et al., 2012), and pasta cooking (Zhang et al., 2008a), which determine the end-use value of durum wheat (Fiedler et al., 2017; N’Diaye et al., 2017). Also, most of those genetic dissection studies conducted in durum wheat were based on bi-parental mapping and low-resolution maps. Genome-wide association mapping studies based on advanced breeding lines may have more relevant results compared with those generated from bi-parental populations and diverse panels because of their direct application in the breeding program.

During the process of developing germplasm and cultivars in a breeding program, a large amount of phenotypic data are routinely collected on breeding lines in advanced generations. However, each year, only a small number of advanced breeding lines are evaluated. A few of those are replaced with new lines over the next few years, resulting in data from a large collection of advanced breeding lines composed of genotypes that are evaluated in different years and locations, generating unbalanced data. With genotyping cost decreasing day by day, the AM approach for identifying QTL could be even more cost-effective if a large amount of phenotypic data routinely collected by breeding programs could be used to gain insight of the genetics of quantitative traits and to identify MTAs for molecular breeding. However, information about the application of such unbalanced data for genetic studies is mostly missing. Therefore, in this study, a panel of advanced breeding lines and cultivars of durum wheat were used to identify 1) genomic regions associated with 24 quality traits, 2) associated markers suitable for MAS, and 3) whether historic unbalanced data are suitable for AM analysis and QTL identification. To our knowledge, this is the first such comprehensive study demonstrating the application of historic unbalanced phenotypic data for genome-wide association studies of complex, low, and moderate heritability traits in wheat.

## Materials and Methods

### Plant Material and Field Evaluation

The AM panel consisted of 243 durum wheat cultivars and inbred lines (F_5:9_) entered into the Uniform Regional Durum Nursery (URDN) from 1997 to 2014 (except in 2010 and 2011 due to severe weather conditions). These cultivars and inbred lines were chosen based on the phenotypic data available for the agronomic and quality traits routinely collected over the years. The historic unbalanced data were collected on these lines from 1997 until 2014. Each year, a subset of those lines was evaluated at five locations in North Dakota (Williston, Minot, Langdon, Carrington, and Prosper) in a randomized complete block design (RCBD) with four replications. Some of the lines were replaced each year with new inbred lines and evaluated in the same manner. So the unbalanced phenotypic data for the panel were obtained for the 80 environments belonging to 16 growing seasons. In 2015, a panel of 256 lines (13 additional lines were added to make a 16 × 16 lattice) was also evaluated together at two locations (Prosper and Langdon) to collect balanced data for the traits under study. Genotypes were planted in a 16 × 16 simple lattice design with two replicates. For both datasets, individual plots consisted of four 2-m-long rows spaced 0.3 m apart. Plots were harvested with a plot combine (HEGE 140), and the grain was collected in individual sacks. The grain was dried to approximately 12% moisture content and stored at 16°C until further processing. Quality tests were performed in the Durum Wheat and Pasta Processing Laboratory at North Dakota State University (NDSU).

### Data Collection

#### Sample Preparation

Post-harvest cleaning was conducted using the Carter-Day Dockage Tester (Simon-Carter-Day Company, Minneapolis, Minnesota) configured with a number 25 riddle and a number 2 top sieve and number 2 bottom sieve. The samples were cleaned again using the Carter-Day Dockage Tester configured with a number 25 riddle, a number 8 top sieve, and a number 2 bottom sieve. For quality traits analyses, subsamples from the total amount of seed available were taken and ground into whole-wheat flour using a Udy Cyclone Mill (UDY Corporation, Boulder, Colorado) fitted with a 60-mesh sieve and stored in plastic bags at 4°C until tests were performed. Ash content, protein content, sedimentation volume, falling number, and total yellow pigment content tests were performed using the whole-wheat samples.

Subsamples for milling were taken from the available grain. Prior to milling, all subsamples were tempered in two stages based on grain moisture. In the first stage, they were tempered to 12.5% moisture for at least 72 h; and in the second stage, they were tempered to 15% moisture for 24 h before milling. The samples were milled into semolina using a Quadramat Jr. Mill (C.W. Brabender Instruments, Inc., South Hackensack, New Jersey) according to the American Association of Cereal Chemists International (AACCI) method 26-50.01 (AACC International, 2008). Semolina samples were kept at 4°C until further analysis. Different approaches for semolina mixing and extrusion were used for the two datasets due to the amount of semolina available. For the historic unbalanced dataset, 1,000 g of semolina was hydrated and mixed in a Hobart C-100-T mixer equipped with a pastry knife agitator. The mixer was set on low speed for 10 s while distilled water was added and then on high speed for 50 s. The semolina was mixed for an additional 2 min on high speed to ensure it reached a complete premix stage. Processing was done in a semicommercial-scale pasta extruder (DeMaco, Melbourne, Florida) and extruded through an 84-strand 0.043-inch Teflon spaghetti die. A jacketed extrusion tube (23-cm length × 4.4-cm inside diameter) was attached to the pasta extruder to allow a longer time for semolina hydration to minimize white specks in the spaghetti. Actual conditions for dough extrusion were a screw rotation speed at 28–29 rpm, a vacuum at 0.8–1.05 kg/cm^2^, and a jacket temperature at 46–48°C. Room temperature and relative humidity were maintained at 25°C and 40–45%, respectively.

For the 2015 balanced dataset, 300 g of semolina was hydrated and mixed in a KitchenAid commercial mixer. The mixer was set on low speed for 10 s while distilled water was added and then on high speed for 50 s. The semolina was mixed for an additional 2 min on high to ensure it reached a complete premix stage. Processing was done using a commercial tabletop electric pasta machine (Arcobaleno, Lancaster, PA, model AEX18) and extruded through a 35-strand 1.09-mm Teflon spaghetti die.

Due to the large number of samples evaluated in 2015 and the amount of time needed for drying, the drying process was omitted for the 2015 balanced dataset. For the historic unbalanced dataset, the extruded spaghetti was dried in a laboratory pilot-scale dryer (Standard Industries, Fargo, North Dakota) on the low-temperature cycle with a total drying time of 18 h. The low-temperature cycle typically has an 18-h total drying time at 40°C (Yue et al., 1999).

Two different spaghetti cooking times were used for the two datasets. In the historic unbalanced dataset, dry spaghetti (10 g) was broken into lengths of approximately 5 cm and placed in 300 ml of boiling water for 12 min. However, based on preliminary results, the cooking time for the fresh spaghetti in the 2015 balanced dataset was reduced to 4 min. Fresh spaghetti (10 g) was cut into lengths of approximately 5 cm and placed in 300 ml of boiling water for 4 min. The optimum cooking time was determined using AACC method 66-50 (AACC International, 2008).

Phenotypic data for following quality traits were recorded:

Protein

*Grain protein content (WPROT)*: Protein content was determined using an Infratec 1226 Whole Grain Analyzer (FOSS Tecator, Höganäs, Sweden). The data on protein content were adjusted to 14% moisture content.
*Semolina protein content (SPROT)*: Semolina protein content was determined using AACC method 39-25.01 (AACC International, 2008) adapted for the FOSS Infratec 1241 Grain Analyzer (Foss North America, Eden Prairie, Minnesota).

Milling-Related Traits

To produce durum wheat semolina and flour, the samples were milled using a Quadramat Jr. Mill (C.W. Brabender Instruments, Inc., South Hackensack, New Jersey) according to AACC method 26-50.01 (AACC International, 2008). Total extraction refers to the portion of the durum wheat kernel that can be milled into flour and semolina. Semolina extraction is only that portion of the wheat kernel that is milled into semolina. For uniformity, all extractions were adjusted to 14% moisture and expressed on constant moisture basis.
*Total extraction (TEXT)*: Total extraction was expressed as a percentage weight per weight (w/w) of semolina and flour from tempered durum wheat samples.
*Semolina extraction (SEXT)*: Semolina extraction was expressed as a percentage weight per weight (w/w) of semolina from tempered durum wheat samples.
*Semolina ash content (SASH), moisture content*: Approved methods (AACC International, 2008) were used to determine ash (method 08-01.01) and moisture content (method 44-15.02).
*Vitreousness (VIT)*: The percentage of vitreous kernels was determined by cutting 100 kernels taken at random transversally with a farinator (grain splitter) and identifying the grains that were not fully vitreous according to the appearance of the sectional areas of the endosperm. Vitreous grains are translucent and transparent when cut, while starchy grains are white and opaque due to the existence of air pockets in the endosperm (Hoseney, 1986).

Gluten-Related Traits

*Sedimentation volume (SDS)*: The sedimentation volume was measured using sodium dodecyl sulfate (SDS) micro-sedimentation test as described by Dick and Quick (1983).
*Mixogram score (MIXO)*: Ten grams of semolina, based on 14% moisture, was weighed. Water was added based on the grain protein content using a formula (*Y* = 1.5*X* + 43.6) described in AACC method 54-40A (2008), where *Y* = amount of water (ml) added to the sample and *X* = protein content at 14% mb. The 10-g bowl mixograph (National Manufacturing, TMCO Division, Lincoln, Nebraska) was used to measure the dough mixing strength of semolina. Mixing tolerance was scored using a scale of 1 (weak) to 8 (strong).
*Wet gluten (WG) and gluten index (GI)*: Wet gluten and gluten index were determined with the glutomatic instrument (Perten Instruments, Springfield, Illinois) using AACC method 38-12.02 (AACC International, 2008).
*Glutork (GLUT)*: Water binding capacity (water bound in wet gluten) was determined with Glutork 2020 (Perten Instruments, Springfield, Illinois) using AACC method 38-12.02 (AACC International, 2008) and expressed as difference between wet and dry gluten content (%).

Color-Related Traits

*Semolina color (color a, color b, and color L)*: Semolina color was determined using the Minolta colorimeter CIEL CR410 (Hunter lab *L*, a, b). Value “*L*” indicates lightness or brightness, value “b” indicates yellowness, and value “a” indicates “greenness.”
  Difference in color a (dif_a), difference in color b (dif_b), and difference in color L (dif_L):
  A semolina dough sheet was made using a modified method described by Fu et al. (2011). A total of 30 g of semolina was hydrated to 38% moisture at 45°C and mixed for 1 min in a KitchenAid mixer (4.3 L KitchenAid CLASSIC Stand Mixer 5K45SS) at speed 4. After being mixed, the dough was sheeted twice in a pair of sheeting rolls with a gap of 1 mm. The resulting dough sheet was folded twice and sheeted twice in a pair of sheeting rolls with a gap of 3 mm without folding. The smooth dough sheet was transferred to a plastic bag and stored in a closed drawer at room temperature. Color was measured on the dough sheet at intervals of 0.5 and 24 h using a Minolta colorimeter CIEL CR410 (Hunter lab *L*, a, b). Differences in color a, color b, and color L were measured between the time intervals.
*Dry pasta color (color)*: Visual color was determined under the constant light source and assigned a numerical visual color score from 1 to 12, with 12 as the best score. The scores were generated according to the color map designed by Debbouz (1994).
*Total yellow pigment (TYP)*: Total yellow pigment (TYP) content was determined using the water-saturated *n*-butanol AACC method 14-50.01 (AACC International, 2008) as modified by using 2 g of ground whole meal. Water-saturated *n*-butanol (10 ml) was added to 2 g of whole meal and shaken for 2 min. After a 30-min rest, the extracts were centrifuged at 12,000 rpm for 10 min, and the supernatant was carefully transferred to cuvettes. Absorbance was measured using a spectrophotometer (Beckman Coulter DU 720 General Purpose UV/Vis Spectrophotometer) at a wavelength of 435.8 nm. Measurements per extracted sample were recorded, and values averaged and converted to yellow pigment concentration (μg/g) using the extinction coefficient (1.6632) for β-carotene (Sims and Lepage, 1968).
*Polyphenol oxidase (PPO)*: Polyphenol oxidase activity was determined using intact kernels as described by Anderson and Morris (2001) using AACC method 22-85.01 (AACC International, 2008). A 1.5 ml aliquot of 10 mM of l-DOPA (l-3,4-dihydroxyphenylalanine) containing 0.02% v/v Tween-20 as a substrate in a 50 mM of MOPS [3-(*N*-morpholino)propane sulfonic acid] buffer with a pH of 6.5 was added to five undamaged seeds in a 2-ml microcentrifuge tube. The tubes were placed on an orbital shaker (Glas-Col, Terre Haute, Indiana) and rotated for 1 h at room temperature to allow the reaction to occur. Polyphenol oxidase activity was measured as the change in absorbance at 475 nm using a Beckman Coulter spectrophotometer (Beckman Coulter DU 720 General Purpose UV/Vis Spectrophotometer, Fullerton, California). The l-DOPA solution was made fresh daily. Each sample was measured in duplicate.

Cooking-Related Traits

*Cooked weight (CWT)*: After being cooked, samples were rinsed off with distilled water in a Buchner funnel and drained. Spaghetti strips were weighed and reported in grams.
*Cooking loss (CLOSS)*: Cooking loss (% weight of solids) was measured by evaporating the cooking water to dryness in a forced-air oven at 110°C overnight. The residue was weighed and reported as percentage of the dry spaghetti.
*Cooked spaghetti firmness (FIRM)*: Cooked firmness was measured using a plexiglass blade probe attached to a Texture Analyzer (Model TA-XT, Texture Technology Corporation, Scarsdale, New York) as described by Walsh (1971). Five strands of cooked spaghetti were placed on a plexiglass plate and sheared at a 90° angle with a plexiglass tooth probe. Firmness was measured as the maximum shear strength of curve (g).
*Work to shear (WS)*: Five strands of cooked spaghetti were placed on a plexiglass plate and sheared at a 90° angle with a plexiglass tooth probe. A TA-XT2 texture analyzer was used to calculate the area under the curve (g·cm), indicating the amount of work required to shear the cooked spaghetti (the CF score). The average of three CF scores was used to report CF.

### Statistical Analysis

The analysis of variance was conducted using the Statistical Analysis System (SAS) computer package version 9.3 (SAS, 2004). The unbalanced historic dataset was analyzed using a mixed linear model (MLM) with Proc Mixed method III, where genotypes were the fixed effects, and environments and replicates within environments were the random effects. The balanced dataset was analyzed using Proc GLM method III. Least square (LS) means were used for the analyses (Steel and Torrie, 1980).

The entry means plot-based heritability for all the traits were estimated using the method described by Holland et al. (2003). The variance and covariance parameters were calculated using the COVTEST and ASYCOV options of the MIXED procedure (SAS, 2004), with environments and genotypes deemed random. Trait correlations were calculated and plotted in R 3.0 (Venables et al., 2017) using cor.matrix and corrplot from the corrplot package. Correlation values were considered significantly different from zero at *P* ≤ 0.05.

### DNA Isolation and SNP-Marker Genotyping and Analysis

Four seeds from each genotype were planted into potting mix in the greenhouse in the fall of 2014. Three young leaf tissues from each genotype were harvested and sent to the USDA-ARS Cereal Crops Genotyping Laboratory in Fargo, ND, for DNA isolation. The extracted DNA samples were genotyped using the Illumina 90k iSelect BeadChip platform, and the markers were called using the diploid version of GenomeStudio software (Wang et al., 2014). FastPHASE 1.3 software with the default settings (Scheet and Stephens, 2006) was used to impute missing loci data using a “likelihood”-based imputation. The heterozygotes were considered missing. Only markers having a minor allele frequency (MAF) > 0.05 were considered for further analysis.

Linkage disequilibrium for all pairwise comparisons between intra-chromosomal SNP was computed, and the genome-wide LD decay was estimated using JMP Genomics 8.1 software (SAS, 2004). The LD was computed as the squared correlation coefficient (*R* ^2^) for each of the marker pairs. Genome-wide LD decay was estimated by plotting LD estimates (*R* ^2^) from all 14 chromosomes against the corresponding pairwise genetic distances (cM). Smoothing spline Fit (lambda = 338064.8) was applied to the estimate of LD decay.

### Association Mapping (AM) Analysis

Association mapping analysis was done using JMP Genomics 8.1 software (SAS, 2004; Zhao et al., 2007). Population structure (Q matrix), which can be defined as the differential relatedness among genotypes, was controlled with principal component analysis (PCA). The identity-by-state (IBS) matrix (K matrix) representing the proportion of shared alleles for all pairwise comparisons in each population was applied. Five regression models were generated to analyze marker–trait association: 1) naive, 2) kinship, 3) kinship plus population structure (the first two principle components (PCs) collectively explained 11.3% of genotype variation), 4) kinship plus population structure (the first three PCs collectively explained 15.46% of genotype variation), and 5) the kinship plus population structure (the first four PCs collectively explained 19.2% of genotype variation). The best model was determined according to the Bayesian information criterion (BIC), where the lowest BIC value is preferred (Ghosh et al., 2006; Zhang et al., 2010). The *P* values of the selected models were later adjusted by calculating the corresponding false discovery rate (FDR) (Benjamini and Yekutieli, 2001). Marker–trait associations were considered significant at an FDR ≤ 0.1.

## Results

### Phenotypic Data Analysis

**Figure 1:**
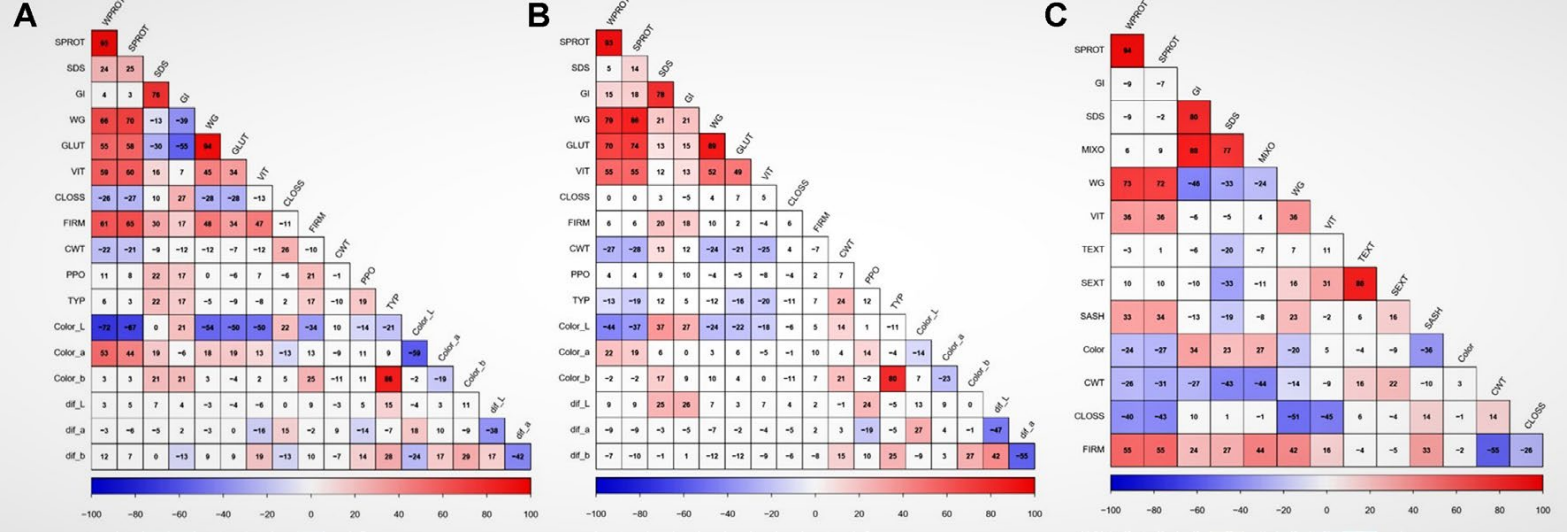
Correlation between traits and locations based on adjusted means. Data showing the relationship between quality traits in **(A)** Prosper, **(B)** Langdon, and **(C)** unbalanced combined data. WPROT, whole-wheat protein; SPROT, semolina protein; SDS, sedimentation test; GI, gluten index; WG, wet gluten; GLUT, glutork; VIT, vitreousness; CLOSS, cooking loss; FIRM, firmness; CWT, cooked weight; PPO, polyphenol oxidase; TYP, total yellow pigment; Color_L, semolina color (Hunter lab value); Color_a, semolina color (Hunter lab value a); Color_b, semolina color (Hunter lab value b); Dif_L, difference in semolina dough sheet color L after 24 h; dif_a, difference in semolina dough sheet color a after 24 h; dif_b, difference in in semolina dough sheet color b after 24 h; Color, spaghetti color; TEXT, total extraction; SEXT, semolina extraction; SASH, semolina ash; MIXO, mixogram score. Cells with correlation values not significant at *P* value < 0.01 have a white background. ## GLUT, WTS, Color_L, Color_a, color_b, Dif_L, Dif_a, Dif_b, PPO, and TYP were only measured in balanced dataset, while TEXT, SEXT, SASH, MIXO, and Color were only measured in historic unbalanced dataset.

There were significant differences among genotypes for most of the traits in both balanced and unbalanced datasets (**Table 1**). Also, the environment had a significant effect on most of the traits as indicated by the significant genotype by environment interactions (**Table 1**). The correlation analysis showed significant correlation between related traits. Grain protein (WPROT) was consistently positively correlated with VIT, WG, FIRM, and GLUT and negatively correlated with CWT and CLOSS (**Figure 1**). Semolina ash (SASH) was positively correlated with WPROT. Total yellow pigment (TYP) was positively correlated with semolina color b and negatively correlated with semolina color L (**Figure 1**). Gluten strength, measured by GI, SDS, and MIXO, was significantly positively correlated with spaghetti firmness (FIRM), but SDS, MIXO, and GI had no significant correlation with WPROT, CWT, or CLOSS (**Figure 1**). These findings suggest that both protein quantity and quality/composition play an independent role in the end-use product as has been observed in the past (Ciaffi et al., 1991; Samaan et al., 2006). Overall, the correlation analysis showed that the protein quantity and quality characteristics were associated with the cooking properties.

**Table 1:** Analysis of variance and heritability estimates for different quality traits in durum wheat association mapping panel.

Traits	Mean squares					H^¶^	SE^#^
	Genotype (G)	Location (L)	Rep(L)	G*L	Error		
**Unbalanced data**							
Grain protein (WPROT)	1.92**	87.19*	88.74**	0.41**	0.35	0.209	0.020
Semolina protein (SPROT)	1.45**	82.62*	79.82**	0.36**	0.27	0.194	0.020
Total extraction (TEXT)	6.96**	546.61**	89.63**	1.24*	1.07	0.288	0.024
Semolina extraction (SEXT)	8.84**	405.46**	79.43**	1.28	1.20	0.338	0.024
Semolina ash (SASH)	0.008**	0.313**	0.233**	0.002**	0.002	0.224	0.022
Kernel vitreousness (VIT)	47,310**	32,988**	300,035**	16,500	152,094	0.223	0.021
Sedimentation volume (SDS)	643.98**	2,636.55**	1,953.89**	28.72**	21.77	0.574	0.023
Gluten index (GI)	3,531.53**	6,918.65**	3,226.44**	116.41**	91.77	0.655	0.021
Wet gluten (WG)	44.04**	766.45*	773.30**	7.69**	5.43	0.291	0.025
Mixogram score (MIXO)	12.09**	8.95	11.54**	0.54**	0.42**	0.551	0.023
Pasta color (Color)	0.62**	14.42**	9.59**	0.12**	0.10	0.272	0.025
Firmness (FIRM)	1.85**	136.52*	135.37**	0.28**	0.22	0.262	0.022
Cooking loss (CLOSS)	0.54**	13.48	18.44**	0.21**	0.16	0.116	0.017
Cooked weight (CWT)	1.87**	37.84**	24.67**	0.72**	0.60	0.088	0.011
**Balanced data**							
Grain protein (WPROT)	0.69**	382.30**	7.05**	0.26**	0.10	0.385	0.043
Semolina protein (SPROT)	0.86**	570.61**	5.66**	0.27**	0.10	0.447	0.042
Kernel vitreousness (VIT)	205.03**	43,076.09**	14,301.21**	72.65**	45.93	0.358	0.040
Sedimentation volume (SDS)	210.49**	9,123.01**	5,242.92**	28.01**	16.62	0.670	0.027
Gluten index (GI)	1,000.44**	405,220.08**	6,920.06**	176.54**	78.81	0.619	0.032
Wet gluten (WG)	18.30**	14,402.79**	235.27**	6.49**	2.16	0.411	0.043
Glutork (GLUT)	0.07**	47.78**	0.61**	0.03**	0.01	0.297	0.056
Color a (Color_a)	0.66**	77.01**	6.09**	0.22**	0.11	0.403	0.041
Color b (Color_b)	11.63**	645.64**	18.65**	1.19**	0.75	0.730	0.023
Color L (Color_L)	2.29**	1,030.62**	13.72**	1.30**	0.75	0.195	0.042
Difference in color a (Dif_a)	0.13**	97.65**	0.38**	0.08*	0.06	0.169	0.038
Difference in color b (Dif_b)	1.90**	219.48**	0.38	0.95**	0.64	0.232	0.041
Difference in color L (Dif_L)	7.45	585.54**	47.02**	7.29	6.60	0.005	0.033
Total yellow pigment (TYP)	3.33**	128.28**	7.16**	0.24**	0.14	0.803	0.018
Polyphenol oxidase activity (PPO)	0.0568**	0.0486**	0.0026	0.0018**	0.0011	0.905	0.009
Firmness (FIRM)	1.98**	135.67*	136.76**	0.28**	0.22	0.456	0.023
Cooking loss (CLOSS)	0.16	52.68**	38.04**	0.18**	0.14	0.103	0.045
Cooked weight (CWT)	0.85	2.79	8.81**	0.85	0.80	0.156	0.044

*,** Significance at P < 0.05 and 0.01, respectively; ns not significant at P < 0.05; G*L, genotype by location interaction; Rep(L), replicates within location; Error, plots residuals; ^¶^broad sense heritability on plot basis calculated for the RILs.

^#^standard error for heritability.

### Marker Properties and Linkage Disequilibrium (LD) Analysis

**Figure 2:**
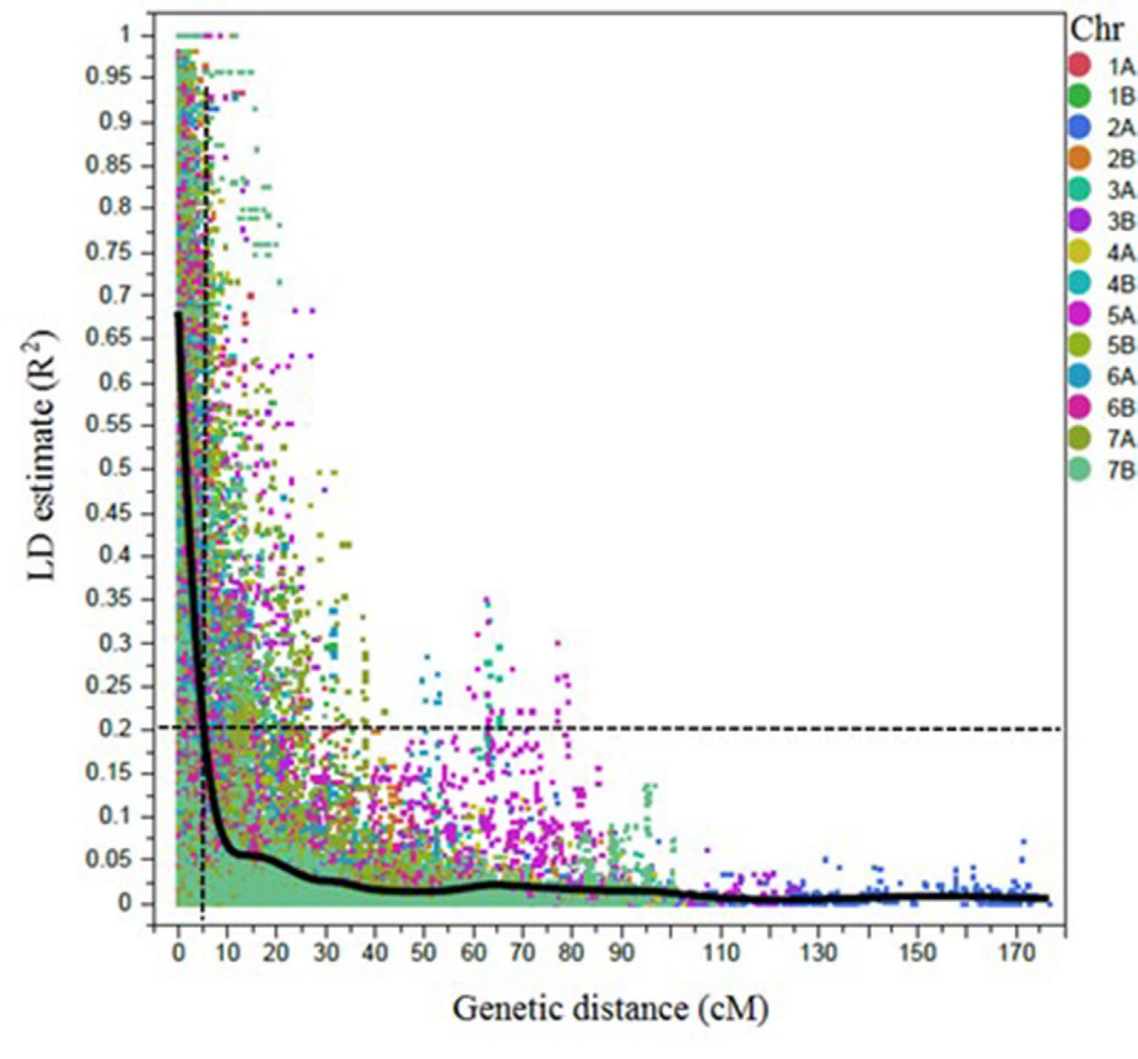
Scatter plot showing the linkage disequilibrium (LD) decay across the chromosomes (Chr) for 243 durum wheat genotypes. The genetic distance in centimorgan (cM) is plotted against the LD estimate (*R* ^2^) for pairs of SNPs. Smoothing spline fit, lambda = 338,064.8, *R* ^2^ = 0.56902, and sum of squares error = 6,804.7.

A total of 6,492 SNP markers showed polymorphism among durum lines (**Supplementary Table 1**). Out of those, 4,196 SNP markers were selected for LD/association mapping (AM) after excluding the markers with MAF <5%, missing data points >10%, and markers with no genetic position on the consensus durum wheat map (Maccaferri et al., 2015). Markers were ordered according to the scaled map positions of the tetraploid wheat SNP consensus map (Maccaferri et al., 2015). The LD decayed to 0.2 within 5 cM, on average (**Figure 2**). Significantly associated SNPs that were ≤5 cM apart and/or located between the pairwise LD (*R* ^2^) ≥ 0.7 were considered a single QTL.

### Population Structure, Kinship Analysis, and Regression Model Selection for AM

**Figure 3:**
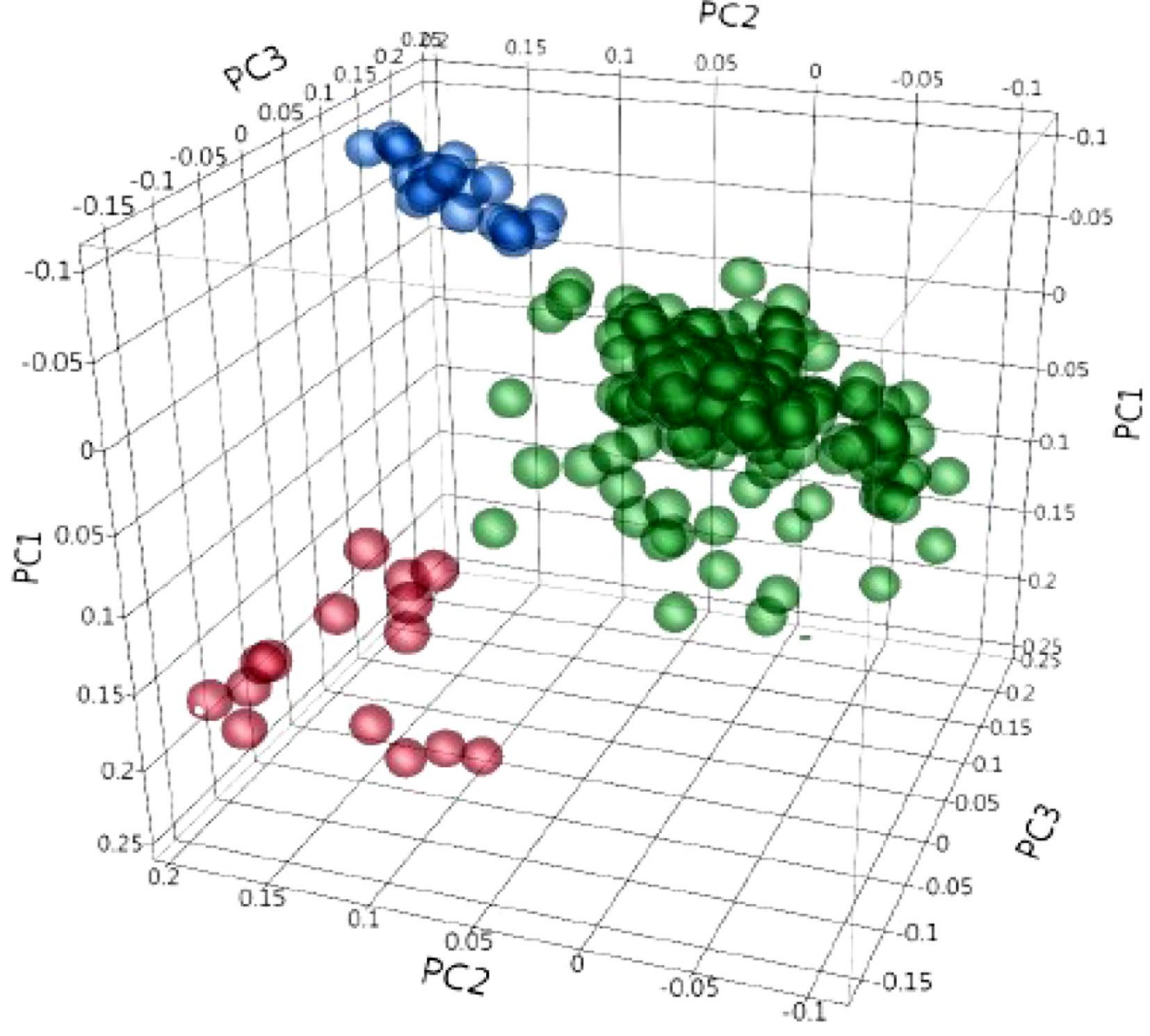
Principal component (PC) analysis obtained from 4,196 polymorphic SNPs, indicating the population structure in 243 durum wheat accessions. PC1, PC2, and PC3 explain 6.8%, 4.5%, and 4.1% of the variation, respectively. The colors represent three different clusters.

**Figure 4:**
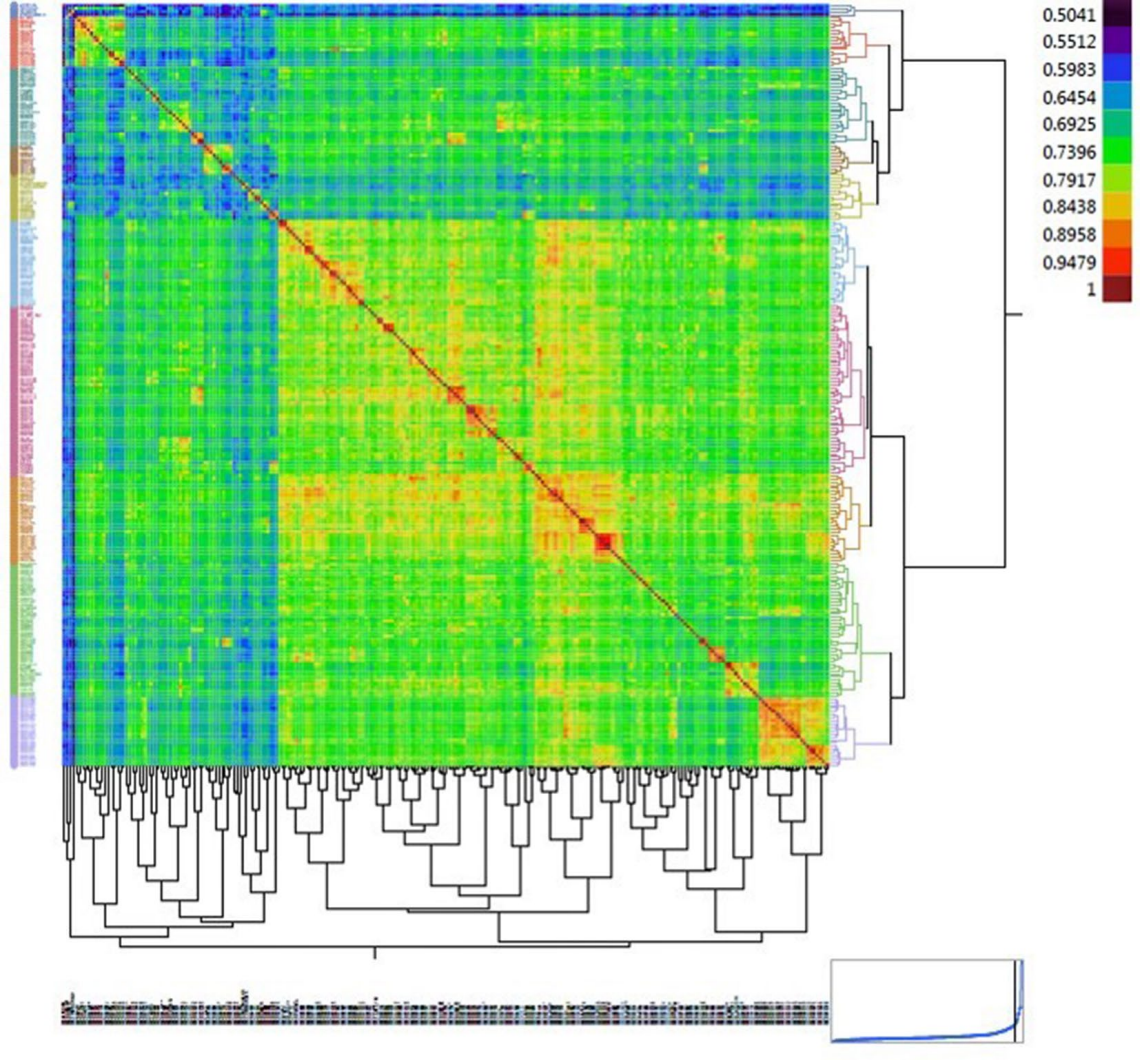
Heat map displaying the relationship matrix among durum wheat genotypes. The red diagonal represents a perfect relationship of each accession with itself. The symmetric off-diagonal elements represent the relationship measures (identity-by-state) for pairs of genotypes. The blocks of warmer colors on the diagonal show clusters of closely related genotypes.

Population structure was inferred using principal component analysis (PCA). Principal component analysis showed that two, three, and 10 PCs explain a cumulative 11.3%, 15.4%, and 26.8% of the genotype variation, respectively. The first three PCs clustered the collection into three sub-populations (**Figure 3**). The familial relatedness was estimated using an identity-by-state matrix (K matrix), and kinship between accessions was calculated (**Figure 4**). Some hotspots with related lines were observed on the heat map, suggesting intermediate familial relationships among genotypes. Accounting for the population structure and familial relationship between individuals in the AM analysis reduces the number of false-positive associations. Based on the BIC values of the five regression models (as explained in the Materials and Methods) tested, no single model fits best for all traits in different environments (**Supplementary Table 2**). For most of the traits, mixed models (KQ) incorporating information about familial relatedness (K matrix) and population structure (Q matrix) were found more suitable (meaning that they have lower BIC values). In some cases of QK model fitting, three PCAs and in some cases four PCAs were found more appropriate. If BIC values for a particular traits were the same using three and four PCAs, we preferred to use three PCAs in the final GWAS model. For few other traits, adding information about population structure in the model did not help. So to avoid any false negatives because of model overfitting, we analyzed those traits with regression models incorporating only familial relatedness (or K matrix).

### Identification of Marker–Trait Associations (MTAs) in Breeding Program Germplasm

Most quality traits were interrelated (**Figure 1**), which was reflected in their significant correlations and co-localization of the associated loci (**Table 2**; **Supplementary Table 3**). The detailed results of marker–trait association are presented in **Supplementary Table 3**, but significant QTLs identified in two or three datasets (except for traits which were analyzed in single environment) are presented in **Table 2**].

**Table 2:** Genetic location of some important (stable) marker–trait associations (MTAs)* for quality traits in durum wheat. Detailed results of marker–trait association for 24 traits are presented in Supplementary Table 3.

Trait/Chr. ##	Position (cM)^a^	Genomic region	Trials^b^	−log_10_ (*P* value)	*R* ^2^¶
**a. Protein-related traits**					
**Grain protein (WPROT)**					
5B	204.7–206.1	61	II^††^, III^††^	2.86	4.4
7A	59.5–62.5	79	II^†^, III^†^	3.02	4.7
7B	62.2–67.3	90	I^††^, III^††^	2.31	3.4
**Semolina protein (SPROT)**					
5B	204.7–206.1	61	I^††^, III^†^	3.14	4.9
6A	111.9–113.5	68	I^††^, II^†^	3.33	5.1
7A	59.5–59.8	79	I^††^, III	3.31	5.2
**b. Milling-related traits**					
**Total extraction (TEXT)**					
2A	145.9	11	III^††^	2.23	3.3
2B	158.3–161.5	24	III	5.14	8.5
4A	25.8	41	III^†^	3.18	5
**Semolina extraction (SEXT)**					
2A	145.8–145.9	11	III^††^	2.99	3.6
2B	161.5	24	III^††^	2.66	4.1
4A	25.2–25.8	41	III^†^	3.1	4.8
**Kernel vitreousness (VIT)**					
2B	181.6–183.1	26	I^††^, III^††^	2.45	3.6
4B	17.7–22.5	46	I, II	5.63	11.6
7A	59.5–62.5	79	I^††^, II^††^	2.84	4.2
**c. Gluten-related traits**					
**Sedimentation volume (SDS)**					
1A	1.3–4.6	1	I^†^, II^†^, III^†^	4.73	7.7
1B	0.3–6.1	4	I^†^, II^†^, III	5.2	8.7
1B	15.2–15.7	5	I^†^, II, III	6.11	10.2
3A	79.5	31	I^††^, II^††^, III^††^	2.86	4.3
3B	75.5–79.1	36	II^††^, III^††^	2.91	4.4
4A	0	40	I^††^, II^†^	3.31	5.1
7B	65.5	90	I^†^, II^††^	3.18	4.9
**Gluten index (GI)**					
1B	0.3–6.1	4	I^†^, II^††^, III^†^	4.3	7.3
1B	15.2–15.7	5	I^†^, II^†^, III	6.59	11
3A	170.1–176.9	32	I^†^, II^††^	3.49	5.1
3B	75.5–86.9	36	I^††^,II^†^, III^††^	3.15	4.8
6A	67.9–72.4	64	I^†^, II^††^, III^††^	3.47	5.4
7B	169.8–175.9	94	I^††^, II^††^, III^††^	2.67	3.3
**Wet gluten (WG)**					
2A	186.2–189.8	14	I^††^, III^††^	2.86	4.3
2B	146.8	22	II^†^, III^†^	3.24	5.3
4B	22.5–28.8	46	II^†^, III^††^	3.48	5.4
7A	59.5	79	I^††^, III^††^	2.69	4.3
**Glutork (GLUT)**					
6A	67.9–72.4	64	I^††^, II^††^	2.65	3.9
**d. Color-related traits**					
Color_a					
4A	139.2–143.7	44	I^†^, II^†^	4.73	7.6
7A	180.3–181.8	88	I^†^, II^††^	3.31	5.1
7B	195.9–196.5	95	I^†^, II^††^	4.37	6.9
**Color_L**					
2A	189.8	14	I^††^, II^††^	2.91	4.4
6A	0.1–3.1	62	I^††^, II^††^	2.28	3.3
**Difference in color b (dif_b)**					
4A	159.5	45	I^†^, II^††^	3.53	5.5
4B	22.5–26.4	46	I^†^, II^††^	3.81	5.1
7B	120.4–123.2	91	I^†^, II^†^	3.16	4.8
7B	138.3–140.4	93	I^†^, II^††^	3.54	5.5
**Difference in color L (dif_L)**					
2B	17.7–19	17	I^†^, II^†^	4.22	6.7
2B	181.6–183.1	26	I^††^, II^††^	2.48	3.7
**Total yellow pigment (TYP)**					
4A	139.2–143.7	44	I^††^, II^††^	2.72	4.1
7A	180.3–181.8	88	I^†^, II^††^	3.22	5
**Polyphenol oxidase activity (PPO)**					
1A	6.6	1	I, II^†^	5.14	8.3
2B	120.2–124.9	21	I^†^, II	6.67	11
3A	170.1–176.9	32	I, II	9.03	15
3A	183.8–184	33	I, II	7.49	12.3
3B	190.4	38	I, II	9.03	15
3B	198.5–205.1	39	I, II	9.03	15
5A	167.1–167.4	57	I^††^, II^†^	3.39	5.2
5B	63.4	59	I^†^, II	5.95	9.7
6A	105.7	67	I^†^, II^†^	3.84	6
6B	27.1	72	I^†^, II^†^	5.15	8.3
**e. Cooking-related traits**					
**Firmness (FIRM)**					
1B	3–8.5	4	II^††^, III^†^	4.93	8.1
3A	7.3–9	27	I^††^, II^††^	2.71	4.1
3B	4.2–7.4	34	I^†^, II^†^	3.75	5.9
7A	180.3–184.1	88	I^††^, II^††^	2.82	4.2
7B	62.2–67.3	90	I^††^, III^††^	2.9	4.5
**Cooking loss (CLOSS)**					
3B	4.2–7.4	34	I^††^, II^†^	3.75	5.9
4A	129.3	43	I^††^, III^††^	2.45	3.6
6A	127.1–130.0	71	II^††^, III^††^	2.56	3.8
**Work to shear (WTS)**					
2B	153.4	23	I^††^, II^††^	2.32	3.4
**Cooked weight (CWT)**					
1B	3–8.5	4	I^†^, III^†^	3.8	6.2
2B	80.6–84	20	I^†^, III^†^	4.77	7.8
3A	7.3–9	27	I^†^, III^††^	3.85	6
3B	4.2–7.4	34	II, III^††^	4.3	7
7B	62.2-67.3	90	I^††^, III^††^	2.47	3.6

^a^cM, marker position on the consensus durum map of Maccaferri et al. (2015).

^b^I = Prosper balanced trial; II = Langdon balanced trial; III = unbalanced combined dataset where an SNP marker was detected above the pFDR value.

*The specific model to used identify significant MTAs for individual traits are reported in **Supplementary Table 2**.

^†^SNP marker that was detected above –log_10_ (P value) of 3 but below the pFDR value in that trial (environment).

^††^SNP marker that was detected above –log_10_ (P value) of 2 but below the pFDR value in that trial (environment).

^¶^R^2^, proportion of phenotypic variation explained by the individual marker.

^##^GLUT, WTS, Color_L, Color_a, color_b, Dif_L, Dif_a, Dif_b, PPO, and TYP were only measured in the balanced dataset, while TEXT, SEXT, SASH, MIXO, and Color were only measured in the historic dataset.

**Protein content:** Both whole grain protein and semolina protein were genetically dissected in this study. Association mapping identified three loci for grain protein and five loci for semolina protein, including two common loci, located one each on 5B and 7A (**Supplementary Table 3**). The QTLs for protein were located in six genomic regions belonging to five different chromosomes (1B, 5B, 6A, 7A, and 7B). All these loci were identified in both balanced and unbalanced datasets. All these loci were associated with minor effects (*R* ^2^ = 3.2–5.2%) on protein content.

**Milling-related traits:** Milling-related traits are represented by total extraction (TEXT), semolina extraction (SEXT), semolina ash (SASH), and kernel vitreousness (VIT). All these traits except VIT were evaluated in the unbalanced dataset only. A total of four, six, eight, and 11 loci were associated with TEXT, SEXT, SASH, and VIT, respectively. These 29 QTL were located in 24 genomic regions belonging to all durum wheat chromosomes except 7B. All the four loci associated with TEXT (located on chromosomes 2A, 2B, 4A, and 5A) were also associated with SEXT (**Supplementary Table 3**) as well, suggesting that those two traits are closely associated. On the other hand, SASH and VIT showed some independent genetic control, as only a single QTL for each of those traits was also found associated with another milling-related trait. However, five out of 11 QTL for VIT were also found associated with one or more color-related traits. Two major QTL (*R* ^2^ > 10%) for kernel vitreousness were identified, one each on chromosomes 4B (17.7–22.2 cM) and 5B (146.14–149 cM). This genomic region on 4B was also found associated with Dif_b, CLOSS, Color, Color_a, and WG. Another QTL on 7A was also associated with WPROT, SPROT, and WG. The phenotypic variation contributed by loci associated with milling-related traits varied from 3% to 16.3%.

**Gluten-related traits:** Five gluten-related traits were genetically dissected in this study. These were sedimentation volume (SDS), gluten index (GI), wet gluten (WG), mixogram score (MIXO), and glutork (GLUT). The number of loci identified for these traits ranged from five (MIXO) to 11 (SDS). The 39 QTLs identified for these five gluten-related traits were located in 25 genomic regions, belonging to 12 different chromosomes (all except 5A and 5B) (**Supplementary Table 3**). Two consistently detected loci (explaining 7% to 11% of the phenotypic variation) associated with multiple gluten-related traits were identified in close proximity on the short arm of chromosome 1B. These loci were associated with SDS, GI, and MIXO and were identified in both balanced and unbalanced datasets. These loci were also associated with other quality traits like cooking weight and pasta firmness. The SNP marker IWB70674 associated with the locus on 1B at 15.2 cM was significant and stable across all locations and datasets. The FDR value was low in both balanced and unbalanced datasets. Another important QTL for gluten strength was identified on chromosome 1A and explained about 7.7% of the phenotypic variation. This locus associated with GI, FIRM, and CWT and was found in both balanced and unbalanced datasets. The SNP marker IWB6234 associated with this locus on chromosome 1A could also be useful for MAS. Based on the information for these two markers, two haplotypes were identified for gluten strength measured by SDS, GI, and MIXO (**Table 3**). Few other consistent QTLs associated independently with one of the gluten traits were also identified on other chromosomes as well (**Supplementary Table 3**).

**Table 3:** Phenotypic means and *t*-test *P* values for lines in the association mapping panel with various combinations of tightly associated markers for gluten strength and PPO activity.

Marker for MAS	Phenotype	Number of genotypes	Phenotypic mean	Range of the phenotypic trait	Actual nucleotide
**IWB70626**					
Unbalanced	High GI haplotype	179	60.2	22.8–92.5	G
	Low GI haplotype	42	43.9	9.2–69.5	T
*t*-test			**6.66236E−06**		
Mean PL*					
	High GI haplotype	191	47.6	12.9–82.8	G
	Low GI haplotype	49	36.7	1.04–64.7	T
*t*-test			**5.44463E−05**		
**IWB6234 and IWB70626**					
Unbalanced	High GI haplotype	14	69.0	50.9–87.6	GC
	Low GI haplotype	35	44.8	9.2–69.5	TT
			**7.45444E−07**		
**IWB70626**					
Unbalanced	High SDS haplotype	187	52.3	39.0–67.4	C
	Low SDS haplotype	47	45.6	25.2–54.6	T
*t*-test			**1.15538E−12**		
Mean PL	High SDS haplotype	190	55.3	38.8–74.8	C
	Low SDS haplotype	49	50.1	23.5–65.5	T
*t*-test			**2.50915E−05**		
					
**IWB6234 and IWB70626**					
Unbalanced	High SDS haplotype	15	58.4	49.4–65.3	GC
	Low SDS haplotype	39	45.0	25.2–50.9	TT
*t*-test			**7.08108E−11**		
Mean PL	High SDS haplotype	15	66.3	57.3–74.8	GC
	Low SDS haplotype	39	49.0	23.5–61.8	TT
			**5.93077E−10**		
**IWB70626**					
Unbalanced	High Mixo score	187	6.5	4.9–8.3	C
	Low Mixo score	47	5.5	2.5–6.6	T
*t*-test			**3.29702E−12**		
IWB6234 and IWB70626					
	High Mixo score	15	6.7	5.8–7.5	GC
	Low Mixo score	39	5.4	2.5–6.6	TT
*t*-test			**2.758E−07**		
					
**IWA5150**					
Mean PL	Low PPO	237	0.11	0.04–0.54	T
	All other lines	2	0.41	0.34–0.48	G
*t*-test			**0.140956287**		
**IWA1488**					
PL					
	Low PPO	235	0.10	0.04–0.54	A
	All other lines	4	0.43	0.038–0.048	G
*t*-test			**0.000149301**		
**IWB69399**					
Mean PL	Low PPO	229	0.10	0.038–0.048	A
	All other lines	10	0.38	0.06–0.54	G
			**2.20191E−05**		
**IWB23604**	Low PPO	231	0.10	0.038–0.490	T
Mean PL	All other lines	8	0.422	0.10–0.54	G
			**0.000360083**		
**IWA1488 + IWB69399 + IWB23604**					
Mean PL	Low PPO	228	0.95	0.038–0.480	AAT
	All other lines	11	0.43	0.10–0.54	GGG
			**7.32631E−07**		
**IWA5150 + IWA1488 + IWB69399 + IWB23604**					
Mean PL	Low PPO	225	0.09	0.04–0.50	TAAT
	All other lines	14	0.40	0.06–0.54	GGGG
*t*-test			**2.56311E−06**		

*PL, mean of data from Prosper and Langdon locations.

**Color-related traits:** Nine color-related traits were measured in the present study, including dough color a, color b, color L before and after 24 h, indicated pigment loss (dif_a, dif_b, and dif_L), dried pasta color (“color”), total yellow pigment (TYP) in whole-wheat flour, and polyphenol oxidase (PPO) in whole wheat flour. “Color” represents spaghetti color and was only measured in the unbalanced dataset. A total of 58 QTLs, located on 41 different genomic regions, belonging to all 14 chromosomes were identified for nine color-related traits measured in both datasets. Ten genomic regions harbored QTL for two to four different color-related traits. For individual color-related traits, the number of loci ranged from four (color_L) to 13 (PPO). Five regions, located one each on chromosomes 3B, 4A, 4B, 6A, and 7A, could be considered important as they were associated with three to four colored-related traits. The QTL on 3B (86.4–89.4 cM) was associated with color_a, dif_b, and TYP. The QTL on 4AL was associated with pasta color, color_a, color_b, dif_b, and TYP. The locus on 4B was associated with pasta color, color_a, pigment loss defined by dif_b, and VIT (**Supplementary Table 3**). Chromosome 6A harbored a genomic region (124–129.4 cM), which was associated with four color-related traits (color_a, color_b, color_L, and dif_b). Another consistent QTL associated with three color-related traits (TYP, col_b, and dif_b) was detected on chromosome 7A at 180–181.8 cM. Co-localized QTLs for TYP and dough color b were identified on chromosomes 2B and 7A. Dough color a, dough color b, pigment loss (measured as difference in dough color b after 24 h), and overall spaghetti color had overlapping QTL on chromosomes 4A, 4B, and 7A.

Markers strongly associated with PPO were identified on chromosomes 1A (IWA5150), 2B (IWA1488), 3A (IWB69399), and 3B (IWB23604) (**Supplementary Table 3**). All SNP markers had low FDR values. The phenotypic variation explained by these color related QTL ranged from 3.1% to 15%.

**Pasta cooking-related traits:** Pasta cooking parameters, such as cooked firmness (FIRM), cooking loss (CLOSS), work to shear (WTS), and cooked weight (CWT), were scored on fresh pasta in the balanced dataset from both Langdon and Prosper locations. In the unbalanced dataset, the same cooking parameters were taken on dry pasta. A total of 45 QTLs located in 32 genomic regions belonging to 13 different chromosomes (except 5B) were identified for four cooking-related traits (**Supplementary Table 3**). The PVE explained by these QTL ranged from 3% to 14%. Four QTLs, located one each on 1B, 2B, 3A, and 3B, explained >10% PV. An important genomic region for cooking-related traits was identified on the short arm of 3B (4.2–7.4 cM). This region was associated with all four cooking-related traits (CWT, CLOSS, WTS, and FIRM) and was identified in both balanced and unbalanced datasets. The PVE for this 3BS locus ranged from 5.9% to 7.0%. Other important regions that were associated with multiple cooking-related traits and detected in more than one dataset were located on 1B (CWT, FIRM, GI, MIXO, and SDS), 2B (CWT and WTS), 3A (CWT and FIRM), 7A (CWT, WTS, and FIRM), and 7B (CWT, FIRM, SDS, and WPROT). The telomeric region on 1BS, which was also found associated with cooking traits FIRM and CWT, also harbors loci for gluten-related traits (GI, MIXO, and SDS). Ten out of 45 QTL were identified in both the balanced and unbalanced datasets. Differences in spaghetti type (dry vs. fresh) during cooking might account for the low number of shared QTLs between the two datasets.

**Distribution and co-localization of marker–trait associations (MTAs or QTLs):** Co-localized or closely linked QTLs may help in improving several traits simultaneously when desirable alleles for each trait are contributed by the same parent. In this study, a total of 179 MTAs were identified for 24 different quality traits. The number of MTAs per chromosome ranged from 3 to 19. The highest number of MTAs were observed for chromosomes 2B and 6A (19 each), followed by 7A (18) and 1B (17). The lowest number of MTAs was observed for chromosomes 1A and 4B (three each). MTAs for different traits were considered in the same genomic region if they were identified at the same position or very close to each other (<1 cM apart) on the durum consensus map from Maccaferri et al. (2015). Based on this classification, these 179 marker–trait associations were located on 95 genomic regions belonging to all 14 durum wheat chromosomes. Durum A-genome chromosomes harbored 90 MTAs located in 50 genomic regions, whereas B-genome chromosomes showed 89 MTAs that represented 45 genomic regions. Among those 95 genomic regions, 53 regions were associated with only a single MTA or QTL each, while the remaining 42 genomic regions harbored multiple co-localized MTAs (**Supplementary Table 3**). The total MTAs associated with those 42 genomic regions were 126, with an average of three MTAs mapped in a particular genomic region. Individually, those regions harbor two to eight MTAs (genomic regions 36 and 88). This means that a major portion of the MTAs or QTLs for different quality traits was co-localized due to either tight linkage or pleiotropy.

**Comparison of GWAS results from balanced and unbalanced datasets:** To the best of our knowledge, there is no study on durum wheat that compares results from GWAS based on unbalanced and balanced phenotypic data. In this study, a total of nine traits, including grain protein (WPROT), semolina protein (SPROT), sedimentation volume (SDS), wet gluten (WG), gluten index (GI), kernel vitreousness (VIT), pasta firmness (FIRM), cooking loss (CLOSS), and cooked weight (CWT), were evaluated in both balanced and unbalanced trials. However, because FIRM, CLOSS, and CWT were measured on dry pasta in the historic unbalanced data and on fresh pasta in the balanced dataset, we decided not to include their comparison here. For the remaining six traits that were measured in both balanced and unbalanced datasets, we observed 40 and 31 marker–trait associations (MTAs), respectively (**Table 4**). A total of 26 (84%) of the MTAs identified in unbalanced dataset were also identified using balanced datasets. Interestingly, for the five traits (except VIT), all the MTAs identified using the unbalanced dataset were also identified in the balanced dataset. For kernel vitreousness, among the six and eight MTAs identified in balanced and unbalanced dataset, only three were common (**Table 4**).

**Table 4:** Number of marker–trait associations (MTAs) identified for different traits using balanced and unbalanced datasets.

Trait	Balanced data	Unbalanced data	Common
Whole grain protein (WPROT)	3	3	3
Semolina protein (SPROT)	4	3	3
Kernel vitreousness (VIT)	6	8	3
Sedimentation volume (SDS)	11	6	6
Gluten index (GI)	8	6	6
Wet gluten (WG)	8	5	5
**Total**	**40**	**31**	**26**

**Important primary candidates for MAS in durum wheat:** Stable and highly significant QTLs are listed in **Table 2**. However, there were some major QTLs identified in this study, which could be the primary target for MAS. For example, markers associated with major QTL for gluten strength on chromosomes 1A and 1B and for PPO activity on chromosomes 1A, 2B, 3A, and 3B could also be excellent primary candidates for MAS in durum wheat breeding programs (**Table 3**). We conducted a *t*-test for markers and alleles significantly associated with increased phenotypic values to determine their possible usefulness for MAS. The lower the *P* value, the more useful the markers (or alleles) and their combination are for MAS. Two haplotypes for gluten strength and five for polyphenol oxidase activity (PPO) were identified (**Table 3**). In the unbalanced and balanced datasets, the two alleles of the marker IWB70626 explained 16.3% and 10.9%, gluten index mean difference. The combination of two markers (IWB70626 and IWB6234) was able to explain the mean difference of 24.8% for the unbalanced dataset. Similarly, for gluten strength measured with SDS, the two haplotypes (IWB6234 and IWB70626) explained 13.4- and 17.3-ml mean difference in SDS. The two alleles for PPO explained 0.35-ppm mean difference for the phenotype.

### Discussion

The majority of the earlier studies that aimed to genetically dissect protein content, gluten strength, and other quality traits in durum wheat were based on bi-parental mapping populations and low-density linkage maps (Jernigan et al., 2017; Kumar et al., 2018). Genetic dissection using association mapping populations provides a more detailed understanding of QTL responsible for the particular phenotype (Gupta et al., 2014; Gupta et al., 2019). Further, genome-wide association mapping studies based on advanced breeding lines offer the additional advantage that gained information about important alleles and associated markers can be directly applied into the breeding program. Also, the deployment of high-density marker systems like Illumina’s iSelect 90k SNP array in genome-wide studies has the tremendous potential to identify tightly linked markers associated with target traits and map QTL/genes more precisely. This study based on advanced breeding lines and 90k SNP Infinium array was used to dissect a large number of quality traits in durum wheat. Therefore, this study, probably provides the most comprehensive knowledge about the genetic architecture of important durum wheat quality traits. The information gained from this study has direct implications for durum wheat breeding using genomics-based tools.

### Genetics of Traits Related to Nutritional Value Enhancement and Grain Quality in Durum Wheat and Identification of some Major Loci

**Protein- and dough-related traits:** The protein- and dough-related characteristics like grain protein (WPROT), semolina protein (SPROT), gluten index (GI), wet gluten (WG), sedimentation volume (SDS), mixogram score (MIXO), and glutograph (GLUT) are considered the most important parameters of pasta quality and thus represent major breeding targets in the durum cultivar development program. QTLs for grain protein were previously identified on all wheat chromosomes (Kulwal et al., 2005; Bogard et al., 2013; Echeverry-Solarte et al., 2015; Kumar et al., 2018). The limited number of QTL for protein content in this study may be due to the low genetic diversity within the genotypes because of the fixation of high-protein-content QTL alleles. The QTL identified on chromosome 7A was located in the same region, which has been reported in earlier studies (Groos et al., 2003; Prasad et al., 2003; Sun et al., 2009). However, QTLs on chromosomes 5B and 7B were not previously reported in durum wheat and may be novel for high grain protein. Our durum germplasm also lacks the major protein content-associated gene *Gpc-B1* (Uauy et al., 2006), thus offering an opportunity to introgress the functional allele of this major gene to enhance grain protein content. Several examples now exist for the successful introgression of this high GPC gene (*Gpc-B1*) into the adapted germplasm, through MAS (Kumar et al., 2011; for review see, Kumar et al., 2018).

Kernel vitreousness (VIT) is also an important characteristic associated with many grain, flour, semolina, and pasta quality traits, including milling and pasta firmness. A high percentage of vitreous kernels maximizes semolina yield (Dexter et al., 1994). Vitreous areas of the endosperm are known to be higher in protein than mealy ones (Matsuo and Dexter, 1980; Dexter et al., 1994). Protein content showed a positive correlation with kernel vitreousness in this study, as has been reported earlier (Sissons 2004; Bilgin et al., 2010; Sieber et al., 2015). The QTL for grain protein that was identified on chromosome 7A (59.5–62.5 cM) was also associated with vitreousness. This locus could help simultaneous improvement in both the traits.

Previous studies have also shown that the high-molecular-weight glutenin subunits (HMW-GS) are particularly important for determining dough elasticity and correlated positively with dough baking quality (Anjum et al., 2007). Group 1 chromosomes harbor genes for glutenin subunits: HMW-GS loci (Glu-A1, Glu-B1, and Glu-D1) on their long arms (Payne and Lawrence, 1983) and LMW-GS loci (Glu-A3, Glu-B3, and Glu-D3) on their short arms (D’Ovidio and Masci, 2004). Also, the QTLs for gluten strength have been identified on most durum and hexaploid wheat chromosomes; the major and most consistent effect across environments is associated with group 1 chromosomes, particularly 1B (Elouafi et al., 2000; Patil et al., 2009; Conti et al., 2011; Kumar et al., 2013). In this study also, two closely located loci on 1BS (0.3–6.1 and 15.2–15.7 cM) have the most significant effects associated with three gluten-related traits (SDS, GI, and MIXO) across environments. Similarly, the homeologous region on 1AS (1.3–4.6 cM) was also associated with multiple traits across different datasets. The association of these two regions with multiple gluten-related traits was expected because of the high correlations between GI, SDS, and MIXO. The regions on 1AS and 1BS were also associated with cooked weight (CWT) and pasta firmness (FIRM), suggesting the importance of these regions not only for gluten strength but for pasta cooking related quality traits as well. Although QTLs for gluten strength parameters on chromosomes 3B and 7B explained minor phenotypic variation (3–6%), they were consistent across environments and datasets. Conti et al. (2011) also reported QTLs for gluten strength on chromosome 3B. The QTLs on chromosomes 6A and 7B were also earlier reported (Patil et al., 2009; Kumar et al., 2013). In the present study, QTLs for wet gluten and glutograph were identified on chromosomes 1B, 2A, 2B, 6A, and 7A. They shared more common QTLs with semolina protein content than does gluten strength (GI, SDS, and MIXO), suggesting the importance of protein quantity and quality for dough strength and pasta production (D’Egidio et al., 1990; Sissons, 2008).

**Milling quality:** The aim of the durum grain milling process is to maximize semolina and minimize flour production through successive steps of grinding and sieving (Posner, 2009). The process is complex as it depends on different factors, such as the moisture content of the grain, impurities and broken durum wheat kernels, the size and texture of the grain, and grain protein content (González, 1995). The identification of a large number of QTLs for milling quality parameters in this study confirms their polygenic nature (Sun et al., 2009; Wu et al., 2015). All QTLs associated with TEXT were also associated with SEXT, suggesting that these two traits measure the same process, which is most likely under the same genetic control. It is also possible that these loci have a pleiotropic effect on both traits. Similar results were reported in other studies (Hessler et al., 2002; Russo et al., 2014) and were also expected based on phenotypic correlation analysis (**Figure 1**). The QTL on chromosome 1B could be the same as the QTL reported by Zhang et al. (2008a). The QTLs on chromosomes 2B and 6B were not previously reported and could be novel.

Semolina ash (SASH), another important milling trait, has complex genetics. A decrease in grain weight always results in higher ash content or lower extraction rates (Breseghello and Sorrells, 2007; Brevis et al., 2010). The QTLs for SASH on chromosomes 1B and 6A could be the same QTL reported in a previous study (Zhang et al., 2008a).

**Color-related traits:** Essentially, all the QTLs for dough color b, a, and total yellow pigment (TYP) had been reported earlier (Parker et al., 1998; Mares and Campbell, 2001; Hessler et al., 2002; Carrera et al., 2007; Pozniak et al., 2007; Zhang and Dubcovsky, 2008b; Garbus et al., 2009; Verlotta et al., 2010; N’Diaye et al., 2017). Major QTLs for yellowness on chromosomes 7A and 7B have been identified earlier in durum wheat (Zhang et al., 2008b). In durum wheat, chromosome 4B is known to harbor *Lpx-B1.1* and *Lpx-B1.2* genes. Previous studies show that deletion of *Lpx-B1.1* is associated with carotenoid pigment degradation during pasta processing (Hessler et al., 2002; Carrera et al., 2007; Garbus et al., 2009; Verlotta et al., 2010). Earlier studies in durum wheat have also reported a linkage between semi-dwarfing gene *Rht-B1b* and *Lpx-B1.1* (Peng et al., 1999; N’Diaye et al., 2017). Similar results were observed in our study where we found a QTL associated with pigment loss on the short arm of chromosome 4B (18.4 to 28.8 cM), a region that harbors a reduced height gene. Markers for pigment loss on chromosome 4B did not show an association with dough color b^†^ and total yellow pigment, confirming that *Lpx-B1.1* deletion has an effect on LOX activity during processing, but not on initial semolina or pasta color (Borrelli et al., 1999; Carrera et al., 2007). Like our study, the distal region on chromosomes 7A and 7B has been found to be associated with total yellow pigment in other tetraploid wheat studies as well (Pozniak et al., 2007; Zhang and Dubcovsky, 2008b). QTLs for color b^†^ and TYP on chromosome 7B were in the same vicinity as the previously reported *Phytoene synthase 1* locus (PSY-B1) (Pozniak et al., 2007).

For pasta color a^†^ (green-red chromaticity), detection of four loci suggests complex genetic control of this trait. Considering the association of chromosome 3B, 4A, and 4B regions with color, col_b, and dif_b, as well as the negative correlation between dough colors a^†^ and b^†^ (*r* = −0.26) and pigment loss as measured by colors a^†^ and b^†^ (*r* = −0.63), a genetic linkage between these two traits could be suggested. This study’s findings and those of N’Diaye et al. (2017) support the undesirable association between pasta redness and pasta yellowness. Therefore, much effort should focus on breaking the LD to facilitate selecting against redness in dough color. The positive correlation between dif_b and dif_L (*r* = 0.31), as well as the negative correlations between dif_L and dif_a (*r* = −0.41) and dif_b and Dif_a (*r* = −0.63), and a single QTL on chromosome 6A associated with col_b and dif_b, may suggest an indirect masking effect of col_a on col_L by directly influencing col_b, especially in semolina dough over time.

Previously reported QTLs for polyphenol oxidase (PPO) activity were independent from other color-related traits, suggesting that their effect on pasta quality is mostly due to a browning reaction rather than the influence of semolina color components (Zhai et al., 2016). The phenotypic and genetic analyses suggested similar findings in our study. Polyphenol oxidase activity did not show any significant correlations with any other color-related trait, and only two (one each on 1A and 7B) out of 13 genomic regions for PPO showed association with other color-related traits. The major QTL for PPO on chromosome 2B was located in a proximate region compared with that in earlier-reported studies (Beecher et al., 2012; Si et al., 2012). Previously reported major QTLs for PPO on chromosome 2A in tetraploid (Watanabe et al., 2006) and hexaploid wheats (Zhang et al., 2005; He et al., 2007; Wang et al., 2009) could not be identified in the present study. The major QTLs for PPO on 3A and 3B seem to be novel and could be attributed to the different sources of germplasm used in this study.

**Cooking-related traits:** Many QTLs for four cooking-related traits (firmness, cooking loss, work to shear, and cooked weight) shared common regions, suggesting a close association between those traits. Two major regions associated with cooking-related traits were also associated with gluten strength on chromosome 1B and whole grain protein on chromosome 7B. Zhang and Dubcovsky (2008b) also reported QTL for mixograph peak height and width near the QTL for firmness and cooking loss on chromosome 1B. Independent QTLs for firmness on chromosomes 6A and 7A suggest that parameters other than protein quantity and quality also affect pasta firmness and cooking loss. For instance, genes responsible for amylose synthesis are reported on chromosome 7A (Miura et al., 1999).

### Application of Unbalanced Historic Phenotypic Data for Genetic Studies and Molecular Breeding

The collection of phenotypic data requires extensive efforts and resources. Breeding programs collect large amounts of phenotypic data from advanced breeding lines every year for selection purposes. In the yield trial stages, the number of such advanced breeding lines tested each year is relatively small, and some of these lines are replaced by other breeding lines, resulting in an unbalanced dataset of advanced breeding lines developed over time. If these historic unbalanced data could be effectively used for genetic studies in crops, they could save significant amounts of resources and provide useful information for molecular breeding of crops. However, not many studies have been conducted to show the utility of such unbalanced historical data for genetic studies in plants. This could be addressed by comparing the results of genetic analysis using structured balanced data and unbalanced datasets. In barley, Wang et al. (2012) evaluated a set of 384 breeding lines to identify QTL for heading date, a highly heritable trait. The study showed that the unbalanced data could be used to identify the three QTLs that were discovered using balanced dataset. However, a careful consideration of population size and experimental design is needed to reduce false-discovery rate, which was higher in case of unbalanced data. While Wang et al., (2012) studied only a single trait with high heritability, most of the target traits in a breeding program show low-to-moderate heritability and complex genetics. In this study, we included more complex traits, having low (grain protein, semolina protein, and kernel vitreousness) to moderate heritability (sedimentation volume, gluten index, and wet gluten) (**Table 1**). Association mapping identified more MTAs using the balanced data (40 for six traits) compared with the unbalanced data (31 for the same six traits). We observed that about 65% of the MTAs identified by balanced data were also detected by historic unbalanced data, suggesting that either balanced data have slightly higher power in QTL detection or that unbalanced historic data might be associated with false-negative MTAs (Type II error). On the other hand, the majority of the MTAs (84%) identified using historic unbalanced data were also detected using balanced dataset, which indicates that historic unbalanced data did not detect false positives as observed by Wang et al. (2012). Our results clearly demonstrate that historic unbalanced data are suitable for genetic studies of both high and low heritable traits. The fact that no false-positive QTLs were detected using unbalanced data for complex and low heritability traits like protein content offers greater prospects of using historic unbalanced data from the breeding program to generate information for molecular breeding of both simple and complex traits. A couple of recent studies have also shown that historical data could be useful for genomic selection as well (Dawson et al., 2013; Rutkoski et al., 2015).

In the last few years, genotyping has become inexpensive, and a large amount of genotypic data can now be generated quickly. Also, an annotated reference sequence of whole wheat genome is now available. In this scenario, our ability to genetically dissect complex traits using routinely collected phenotypic data by the breeders will be extremely promising. This could help us save resources spent on genetic studies and may enable us to speed up genetic gain through molecular breeding tools.

## Highlights

Multi-environment phenotypic data and high-density SNP platform were used to identify markers associated with 24 nutritional value enhancement and quality traits for genomics-assisted durum wheat breeding. The study also showed the application of historic unbalanced phenotypic data for genetic studies.

## Conclusion

The study aimed to dissect the genetics of durum wheat quality and identify useful marker–trait association for 24 different traits using high-density 90k Infinium SNP marker data. Genome-wide association studies revealed that MTAs for durum quality traits are distributed across the whole genome. Markers associated with some major QTL for gluten strength on chromosomes 1A and 1B and PPO activity on chromosomes 1A, 2B, 3A, and 3B could also be excellent easy candidates for MAS in durum wheat breeding programs. The information gained on extensive genetic dissection of durum wheat quality traits and the resources developed in this study may prove extremely useful to assess quality in early generations by incorporating molecular breeding tools in the breeding program. Another main objective of this study was to explore the possibilities of using a large amount of multi-year, multi-location unbalanced historical data generated by the breeding programs for genetic studies. A large number of common SNPs detected in both the unbalanced historic and balanced datasets suggest that the unbalanced data collected by the plant breeding programs over space and time could be used to gain knowledge about the genetics of important traits and identify MTAs for molecular breeding. This would save huge amounts of resources invested on conducting genetic studies using specifically designed populations. Also, as this study was based on advanced breeding lines, the MTAs identified in this study are easily accessible and should provide more directly useful information for local-knowledge-based breeding.

## Author Contributions

MJ, FM, and EE conceived and designed the experiments. MJ, AO-A, and ES performed the experiment. MJ, AK, and MA analyzed the data. MJ and AK wrote the manuscript with inputs from all the co-authors.

## Conflict of Interest Statement

The authors declare that the research was conducted in the absence of any commercial or financial relationships that could be construed as a potential conflict of interest.

## Abbreviations

AFLP, amplified fragment length polymorphism; AM, association mapping; CLOSS, cooking loss; CV, coefficient of variance; CWT, pasta cooked weight; FIRM, pasta firmness; FN, falling number; GI, gluten index; GWAS, genome-wide association study; LD, linkage disequilibrium; MAF, minor allele frequency; MAS, marker-assisted selection; MIXO, mixogram score; MSD, mean squared difference; PCA, principal component analysis; FDR, false discovery rate; RFLP, restriction fragment length polymorphism; SASH, semolina ash; SDS, sodium dodecyl sulfate micro-sedimentation; SEXT, semolina extraction; SNP, single-nucleotide polymorphism; SPROT, semolina protein; SSR, simple sequence repeat; TEXT, total extraction; TYP, total yellow pigment; WG, wet gluten; WPROT, whole-wheat protein; WTS, work to shear.
