# Supplementary material for: Association Mapping for 24 Traits Related to Protein Content, Gluten Strength, Color, Cooking, and Milling Quality Using Balanced and Unbalanced Data in Durum Wheat [Triticum turgidum L. var. durum (Desf).]
Source: Front Genet. 2019 Aug 16;10:717. doi: 10.3389/fgene.2019.00717 (PMC6706462; doi:10.3389/fgene.2019.00717)
Supplement: Supplementary file 2 [file Table_2.docx]

**Supplementary Table 2** Bayesian information criterion (BIC) value for each model.

| Trait | Naïve | Kinship | 2PCs + Kinship | 3PCs + Kinship | 4PCs +Kinship |  |
| --- | --- | --- | --- | --- | --- | --- |
| **Langdon, 2015** | | | | | |  |
| Grain protein (WPROT) | 320.2 | 309.3 | 306.5 | 306.3 | **297.3** |  |
| Semolina protein (SPROT) | 382.6 | 371.6 | 364.9 | 364.8 | **353.9** |  |
| Kernel vitreousness (VIT) | 1507.5 | 1496.6 | 1481.6 | **1435.2** | 1471.3 |  |
| Sedimentation volume (SDS) | 1674.3 | 1663.4 | 1643.1 | **1605.4** | 1639.9 |  |
| Gluten index (GI) | 2040.8 | 2029.8 | 2001.1 | 2000.4 | **1997.9** |  |
| Wet gluten (WG) | 1089.5 | 1078.6 | 1070 | 1066.8 | **1053.8** |  |
| Glutork (GLUT) | -203.2 | -214.2 | -223.4 | -224.1 | **-228.7** |  |
| Firmness (FIRM) | 1978.8 | 1967.9 | 1965.4 | 1964.8 | **1958** |  |
| Cooking loss (CLOSS) | 90.7 | 79.7 | 78.4 | 77.8 | **70.2** |  |
| Work to shear (WTS) | 784.2 | 773.3 | 770.5 | 770.4 | **767.5** |  |
| Cooked weight (CWT) | 151.3 | 140.3 | 136.4 | 134.7 | **126.6** |  |
| Color a (Color_a) | 225.6 | 214.7 | 207.2 | 202.6 | **191.6** |  |
| Color b (Color_b) | 976.7 | 924.2 | 944.4 | **920.7** | 931.6 |  |
| Color L (Color_L) | 564.8 | 553.8 | 549.4 | 548.5 | **548.3** |  |
| Difference in color a (Dif_a) | -25.2 | -36.1 | -39 | -39 | **-42.7** |  |
| Difference in color b (Dif_b) | 665.2 | 654.3 | 642.4 | 642.1 | **640** |  |
| Difference in color L (Dif_L) | 807.4 | 796.5 | 794.3 | 794.3 | **793** |  |
| Total yellow pigment (TYP) | 614.8 | 529.1 | 551.1 | 551.1 | **519.7** |  |
| Polyphenol oxidase activity (PPO) | -360.5 | -371.4 | -377.8 | -379.2 | **-379.4** |  |
| **Prosper, 2015** | | | | | |  |
| Grain protein (WPROT) | 354.9 | **271.7** | 321.6 | 319.8 | 319.8 | |
| Semolina protein (SPROT) | 383.3 | **282.3** | 353.6 | 352.4 | 352.4 | |
| Kernel vitreousness (VIT) | 1799.3 | 1788.3 | 1773.2 | **1772.1** | 1772.1 | |
| Sedimentation volume (SDS) | 1634.2 | 1623.3 | 1613.2 | **1611.8** | 1611.8 | |
| Gluten index (GI) | 2055.6 | 2044.7 | **1975.5** | 1975.5 | 1975.5 | |
| Wet gluten (WG) | 1121 | 1110 | 1109.5 | **1107.1** | 1107.1 | |
| Glutork (GLUT) | -207.2 | **-256.2** | -219.6 | -221.3 | -221.3 | |
| Firmness (FIRM) | 2119.1 | 2108.1 | 2100.4 | **2100.3** | 2100.3 | |
| Cooking loss (CLOSS) | 104.3 | 93.4 | 91.6 | **90.2** | 90.2 | |
| Work to shear (WTS) | 967.4 | 956.5 | **946.7** | 946.7 | 946.7 | |
| Cooked weight (CWT) | 630.2 | 619.2 | 619.2 | **619** | 619 | |
| Color a (Color_a) | 381.6 | 370.6 | 369 | **364.9** | 364.9 | |
| Color b (Color_b) | 961.9 | 884.3 | 899.6 | **876.3** | 876.3 | |
| Color L (Color_L) | 730.1 | 719.1 | 716.7 | **716.5** | 716.5 | |
| Difference in color a (Dif_a) | -57.6 | -68.5 | -69.8 | **-74.9** | -74.9 | |
| Difference in color b (Dif_b) | 542.9 | 531.9 | 518.4 | **517.3** | 517.3 | |
| Difference in color L (Dif_L) | 472.3 | 461.3 | 456.4 | **455** | 455 | |
| Total yellow pigment (TYP) | 679.5 | **593** | 600.3 | 600.2 | 600.2 | |
| Polyphenol oxidase activity (PPO) | -299.6 | **-337.5** | -321.2 | -321.5 | -321.5 | |

**S Table 2** Bayesian information criterion (BIC) value for each model (continued).

| Trait | Naïve | Kinship | 2PCs + Kinship | 3PCs + Kinship | 4PCs +Kinship |
| --- | --- | --- | --- | --- | --- |
| **Unbalanced combined data from 1997-2014** | | | | | |
| Grain protein (WPROT) | 217.5 | 206.5 | 205.5 | 205.1 | **204.2** |
| Semolina protein (SPROT) | 156.5 | 145.6 | 143.4 | 143.4 | **143.2** |
| Total extraction (TEXT) | 544.2 | 533.3 | 533.2 | **531.6** | 531.6 |
| Semolina extraction (SEXT) | 603.9 | 593 | 590.3 | 590.3 | **590.2** |
| Semolina ash (SASH) | -999.3 | -1010.2 | -1013.9 | -1013.9 | **-1014** |
| Kernel vitreousness (VIT) | 1323.5 | 1312.6 | 1293.2 | 1292.6 | **1292.1** |
| Sedimentation volume (SDS) | 1498.5 | 1487.5 | 1480.6 | 1480.3 | **1477.5** |
| Gluten index (GI) | 1823.3 | 1812.5 | 1729 | 1787.2 | **1786.7** |
| Mixogram score (MIXO) | 545.6 | 449.1 | 508.7 | 507.4 | **446.3** |
| Pasta color (Color) | 40.2 | 29.3 | 20.8 | 14.6 | **-33.5** |
| Firmness (FIRM) | 159.6 | 148.7 | 142.3 | 130.8 | **90.4** |
| Cooking loss (CLOSS) | -29.5 | -40.4 | -69.4 | -83.2 | **-84.2** |
| Cooked weight (CWT) | 150.7 | 139.8 | 136.1 | 135.2 | **123.2** |
| **Mean Langdon + Prosper** | | |  |  |  |
| Grain protein (WPROT) | 263.2 | 252.3 | 247.6 | 232.4 | **231.3** |
| Semolina protein (SPROT) | 325.4 | 314.4 | 311.1 | 295.6 | **293.8** |
| Kernel vitreousness (VIT) | 1622.9 | 1611.9 | 1602.7 | **1591.5** | 1591.5 |
| Sedimentation volume (SDS) | 1623.2 | 1612.3 | 1606.6 | 1593.2 | **1592.6** |
| Gluten index (GI) | 2007.7 | 1996.7 | 1981.9 | **1967.7** | 1967.7 |
| Wet gluten (WG) | 1038.4 | 1027.4 | 1017.7 | 1016.5 | **1012.6** |
| Glutork (GLUT) | -292.7 | -303.7 | -311.6 | -313.5 | **-315.8** |
| Firmness (FIRM) | 1940.5 | 1929.5 | 1919.5 | 1917.8 | **1910.6** |
| Cooking loss (CLOSS) | -85.8 | -96.8 | -98.1 | -98.1 | **-101.8** |
| Work to shear (WTS) | 754.2 | 743.2 | 733.7 | 727.6 | **723.8** |
| Cooked weight (CWT) | 328.7 | 317.7 | 317 | 316.8 | **315.9** |
| Color a (Color_a) | 246.2 | 235.3 | 217.6 | 209.6 | **198.1** |
| Color b (Color_b) | 947 | 936 | 905 | 897.9 | **876.7** |
| Color L (Color_L) | 559.8 | 548.9 | 544.7 | **544** | 544 |
| Difference in color a (Dif_a) | -141.8 | -152.8 | -157.1 | -157.1 | **-157.4** |
| Difference in color b (Dif_b) | 517.8 | 506.9 | 486.2 | 485.3 | **485.1** |
| Difference in color L (Dif_L) | 545.7 | 534.8 | 527.2 | 525.8 | **525.4** |
| Total yellow pigment (TYP) | 633.2 | **542.7** | 577.8 | 569.4 | 560.3 |
| Polyphenol oxidase activity (PPO) | -334.9 | -345.9 | -357.6 | -358.5 | **-358.7** |

^†^2PC, population structure matrix (Q matrix) based on the first two principal components;

3PC, population structure matrix (Q matrix) based on the first three principal components.

^†^Numbers in **bold** indicate the lowest BIC and best model for each trait. The best model was used to investigate single-nucleotide polymorphism–trait associations.
