# Supplementary material for: Association Mapping for 24 Traits Related to Protein Content, Gluten Strength, Color, Cooking, and Milling Quality Using Balanced and Unbalanced Data in Durum Wheat [Triticum turgidum L. var. durum (Desf).]
Source: Front Genet. 2019 Aug 16;10:717. doi: 10.3389/fgene.2019.00717 (PMC6706462; doi:10.3389/fgene.2019.00717)
Supplement: Supplementary file 3 [file Table_3.docx]

| **Supplementary Table 3** Detailed list of significant marker-trait associations* (MTAs) for 24 quality traits in durum wheat, identified in this study. | | | | | | |
| --- | --- | --- | --- | --- | --- | --- |
| **Trait/Chr.** ## | **Position (cM) ^a^** | **Genomic region** | **Other associated traits** | **Trials^b^** | **-log_10_ (P-**  **value)** | **R^2^¶** |
| **a.        Protein related traits** | | | | | | |
| **Grain protein (WPROT)** | |  |  |  |  |  |
| 5B | 204.7-206.1 | 61 | SPROT | II^††^, III^††^ | 2.86 | 4.4 |
| 7A | 59.5-62.5 | 79 | SPROT, WG, VIT | II^†^, III^†^ | 3.02 | 4.7 |
| 7B | 62.2-67.3 | 90 | SDS, FIRM, CWT | I^††^, III^††^ | 2.31 | 3.4 |
| **Semolina protein (SPROT)** | |  |  |  |  |  |
| 1B | 109.0-111.7 | 9 | Dif_A | I^††^, III^††^ | 2.11 | 3.2 |
| 5B | 204.7-206.1 | 61 | WPROT | I^††^, III^†^ | 3.14 | 4.9 |
| 6A | 111.9-113.5 | 68 | FIRM, WG | I^††^, II^†^ | 3.33 | 5.1 |
| 7A | 59.5-59.8 | 79 | WPROT, WG, VIT | I^††^, III | 3.31 | 5.2 |
| 7A | 114 | 84 |  | II^††^, III^††^ | 2.64 | 3.3 |
| **b.       Milling related traits** | | | | | | |
| **Total extraction (TEXT)** | |  |  |  |  |  |
| 2A | 145.9 | 11 | SEXT, CWT | III^††^ | 2.23 | 3.3 |
| 2B | 158.3-161.5 | 24 | SEXT | III | 5.14 | 8.5 |
| 4A | 25.8 | 41 | SEXT, Color | III^†^ | 3.18 | 5 |
| 5A | 127.5-128.3 | 53 | SEXT | III^††^ | 2.07 | 3 |
| **Semolina extraction (SEXT)** | |  |  |  |  |  |
| 2A | 145.8-145.9 | 11 | TEXT, CWT | III^††^ | 2.99 | 3.6 |
| 2B | 161.5 | 24 | TEXT | III^††^ | 2.66 | 4.1 |
| 4A | 25.2-25.8 | 41 | TEXT, Color | III^†^ | 3.1 | 4.8 |
| 5A | 127.5-128.3 | 53 | TEXT | III^††^ | 2.25 | 3.3 |
| **Semolina ash (SASH)** | |  |  |  |  |  |
| 1A | 102.8-105.5 | 3 |  | III^††^ | 2.17 | 3.2 |
| 4A | 39 | 42 |  | III^†^ | 3.13 | 4.9 |
| 5B | 0.9-6.5 | 58 | VIT | III^††^ | 2.45 | 3.7 |
| 6A | 45.2 | 63 |  | III^†^ | 3.12 | 4.9 |
| 6A | 117.7-118.2 | 69 |  | III^†^ | 3.1 | 4.8 |
| 6B | 131.8-135.2 | 77 |  | III^††^ | 2.11 | 3.1 |
| 7A | 70.7 | 80 |  | III^††^ | 2.23 | 3.3 |
| 7A | 136.4 | 86 |  | III^††^ | 2.06 | 3.3 |
| **Kernel vitreousness (VIT)** | |  |  |  |  |  |
| 1B | 88.2-93.5 | 8 | DIF_A | II^††^, III^††^ | 2.51 | 3.8 |
| 1B | 150.9-152.5 | 10 | PPO | I^††^. III^††^ | 2.3 | 3.4 |
| 2B | 181.6-183.1 | 26 | Dif_L, GLUT, WG | I^††^, III^††^ | 2.45 | 3.6 |
| 3B | 100.15-100.9 | 37 |  | I^††^, II^††^ | 2.54 | 3.8 |
| 4B | 17.7-22.5 | 46 | Dif_b, CLOSS, Color, Color_a, WG | I, II | 5.63 | 11.6 |
| 7A | 59.5-62.5 | 79 | WPROT, SPROT, WG | I^††^, II^††^ | 2.84 | 4.2 |
| **c.        Gluten related traits** | | | | | | |
| **Sedimentation volume (SDS)** | |  |  |  |  |  |
| 1A | 1.3-4.6 | 1 | GI, FIRM, CWT, PPO, Color_a | I^†^, II^†^, III^†^ | 4.73 | 7.7 |
| 1A | 48.1-49.7 | 2 |  | I^††^, II^††^ | 2.51 | 3.7 |
| 1B | 0.3-6.1 | 4 | CWT, FIRM, GI, MIXO | I^†^, II^†^, III | 5.2 | 8.7 |
| 1B | 15.2-15.7 | 5 | CWT, GI, MIXO | I^†^, II, III | 6.11 | 10.2 |
| 2B | 169.3-170.9 | 25 | Dif_a | I^††^, III^††^ | 2.49 | 3.7 |
| 3A | 79.5 | 31 |  | I^††^, II^††^, III^††^ | 2.86 | 4.3 |
| 3B | 51.9-56.9 | 35 |  | I^††^, II^††^ | 2.77 | 4.2 |
| 3B | 75.5-79.1 | 36 | GI, MIXO, FIRM, CWT, TYP, Color_a, Dif_b | II^††^, III^††^ | 2.91 | 4.4 |
| 4A | 0 | 40 |  | I^††^, II^†^ | 3.31 | 5.1 |
| 6A | 67.9-72.4 | 64 | GI, CLOSS, GLUT | I^††^, II^††^ | 2.62 | 3.9 |
| 7B | 65.5 | 90 | WPROT, FIRM, CWT | I^†^, II^††^ | 3.18 | 4.9 |
| **Gluten index (GI)** | |  |  |  |  |  |
| 1A | 1.3-4.6 | 1 | SDS, FIRM, CWT, PPO, Color_a | II^†^ | 3.67 | 5.7 |
| 1B | 0.3-6.1 | 4 | CWT, SDS, FIRM, MIXO | I^†^, II^††^, III^†^ | 4.3 | 7.3 |
| 1B | 15.2-15.7 | 5 | CWT, SDS, MIXO | I^†^, II^†^, III | 6.59 | 11 |
| 2A | 186.2-189.8 | 14 | WG, Color_L | I^††^, III^††^ | 2.47 | 3.9 |
| 3A | 170.1-176.9 | 32 | CWT, PPO | I^†^, II^††^ | 3.49 | 5.1 |
| 3B | 75.5-86.9 | 36 | SDS, FIRM, CWT, MIXO, TYP, Color_a, Dif_b | I^††^,II^†^, III^††^ | 3.15 | 4.8 |
| 6A | 67.9-72.4 | 64 | SDS, CLOSS, GLUT | I^†^, II^††^, III^††^ | 3.47 | 5.4 |
| 7B | 169.8-175.9 | 94 | MIXO | I^††^, II^††^, III^††^ | 2.67 | 3.3 |
| **Wet gluten (WG)** | |  |  |  |  |  |
| 2A | 186.2-189.8 | 14 | GI, Color_L | I††, III†† | 2.86 | 4.3 |
| 2B | 146.8 | 22 |  | II†, III† | 3.24 | 5.3 |
| 2B | 181.6-183.1 | 26 | GLUT, VIT, Dif_L | I | 5.17 | 8.3 |
| 4B | 22.5-28.8 | 46 | Color, Color_a, Dif_b, CLOSS, VIT | II†, III†† | 3.48 | 5.4 |
| 4B | 60 | 47 |  | I††, III†† | 2.48 | 3.7 |
| 6A | 112.9 | 68 | SPROT, FIRM | II† | 3.8 | 6 |
| 6B | 155.9 | 78 | GLUT | II† | 4.28 | 6.8 |
| 7A | 59.5 | 79 | SPROT, WPROT, VIT | I††, III†† | 2.69 | 4.3 |
| **Mixogram score (MIXO)** | |  |  |  |  |  |
| 1B | 0.3-6.1 | 4 | CWT, FIRM, GI, SDS | III | 5.72 | 9.5 |
| 1B | 15.2-15.7 | 5 | CWT, GI, SDS | III | 5.64 | 9.3 |
| 2A | 197.6 | 15 |  | III^††^ | 2.49 | 3.8 |
| 3B | 75.5-86.9 | 36 | GI, SDS, FIRM, CWT, TYP, Color_a, Dif_b | III^††^ | 2.49 | 3.7 |
| 7B | 169.8-173.1 | 94 | GI | III^†^ | 3.51 | 5.6 |
| **Glutork (GLUT)** |  |  |  |  |  |  |
| 2A | 169.3-171 | 13 |  | I^††^, II^††^ | 2.67 | 4 |
| 2B | 181.6-183.1 | 26 | WG, VIT, Dif_L | I^†^ | 4.56 | 7.3 |
| 6A | 3-6.6 | 62 | Color_L | I^††^, II^††^ | 2.51 | 3.7 |
| 6A | 67.9-72.4 | 64 | SDS, GI, CLOSS | I^††^, II^††^ | 2.65 | 3.9 |
| 6B | 155.9 | 78 | WG | II^†^ | 3.04 | 4.6 |
| **d.       Color related traits** | | | | | | |
| **Color_a** | |  |  |  |  |  |
| 1A | 6.6 | 1 | SDS, GI, FIRM, PPO, CWT | II^††^ | 2.69 | 4 |
| 3A | 20.9 | 28 | CLOSS | II^†^ | 3.31 | 5.1 |
| 3B | 86.4-88.4 | 36 | TYP, Dif_b, CWT, FIRM, SDS, GI, MIXO | I | 5.65 | 9.2 |
| 4A | 139.2-143.7 | 44 | Color_b, Color, TYP | I^†^, II^†^ | 4.73 | 7.6 |
| 4B | 22.5-26.4 | 46 | Color, Dif_b, VIT, WG, CLOSS | I^†^ | 3.64 | 5.7 |
| 6A | 124-125.6 | 70 | Color_L, Dif_b | I^†^ | 4.51 | 7.2 |
| **Color_b** | |  |  |  |  |  |
| 2B | 6.6-8.3 | 16 | TYP | II^††^ | 2.14 | 3.1 |
| 4A | 139.2-143.7 | 44 | Color_a, Color, TYP | II^††^ | 2.5 | 3.7 |
| 5A | 52.9 | 51 |  | I^††^, II^††^ | 2.46 | 3.6 |
| 6A | 129.4 | 71 | CLOSS | I^††^, II^††^ | 2.5 | 3.7 |
| 7A | 180.3-181.8 | 88 | Dif_L, TYP, FIRM, CWT, WTS | I^†^, II^††^ | 3.31 | 5.1 |
| 7B | 195.9-196.5 | 95 | TYP | I^†^, II^††^ | 4.37 | 6.9 |
| **Color_L** | |  |  |  |  |  |
| 2A | 189.8 | 14 | GI, WG | I^††^, II^††^ | 2.91 | 4.4 |
| 6A | 0.1-3.1 | 62 | GLUT | I^††^, II^††^ | 2.28 | 3.3 |
| 6A | 124.8 | 70 | Color_a, Dif_b | I^†^ | 3.25 | 5 |
| **Difference in color a (dif_a)** | |  |  |  |  |  |
| 1B | 87.8-89.1 | 8 | VIT | II^†^ | 3.7 | 5.8 |
| 1B | 109-111.7 | 9 | SPROT | II^†^ | 3.02 | 4.6 |
| 2B | 171.1 | 25 | SDS | I^†^ | 3.33 | 5.1 |
| 5A | 26.2-27.9 | 50 | Dif_b | II^†^ | 4.1 | 6.6 |
| **Difference in color b (dif_b)** | |  |  |  |  |  |
| 3B | 86.4-89.4 | 36 | Color_a, TYP, CWT, FIRM, SDS, GI, MIXO | I^††^, II^††^ | 2.29 | 3.3 |
| 4A | 159.5 | 45 |  | I^†^, II^††^ | 3.53 | 5.5 |
| 4B | 22.5-26.4 | 46 | VIT, CLOSS, WG, Color_a, Color | I^†^, II^††^ | 3.81 | 5.1 |
| 4B | 115.5 | 48 | CLOSS | I^††^, II^††^ | 2.87 | 4.3 |
| 5A | 26.2-26.5 | 50 | Dif_a | I^††^, II^††^ | 2.42 | 3.6 |
| 6A | 124.8-126.5 | 70 | Color_a, Color_L | I^†^ | 3.23 | 5 |
| 7B | 120.4-123.2 | 91 |  | I^†^, II^†^ | 3.16 | 4.8 |
| 7B | 138.3-140.4 | 93 |  | I^†^, II^††^ | 3.54 | 5.5 |
| **Difference in color L (dif_L)** | |  |  |  |  |  |
| 2B | 17.7-19 | 17 |  | I^†^, II^†^ | 4.22 | 6.7 |
| 2B | 181.6-183.1 | 26 | VIT, GLUT, WG | I^††^, II^††^ | 2.48 | 3.7 |
| 7A | 184.1 | 88 | Color_b, TYP, FIRM, CWT, WTS | II^†^ | 3.16 | 4.8 |
| 7B | 13.8 | 89 | PPO | II^†^ | 3.69 | 5.8 |
| **Pasta color (color)** | |  |  |  |  |  |
| 4A | 23.7-25.6 | 41 | TEXT, SEXT | III^†^ | 3.04 | 4.7 |
| 4A | 139.2-143.7 | 44 | Color_a, Color_b, TYP | III^†^ | 3.56 | 5.6 |
| 4B | 22.5-26.4 | 46 | Color_a, Dif_b, VIT, CLOSS, WG | III^†^ | 3.25 | 5.1 |
| **Total yellow pigment (TYP)** | |  |  |  |  |  |
| 2B | 6.6-8.3 | 16 | Color_b | I^††^, II^††^ | 2.27 | 3.4 |
| 3B | 86.4-88.4 | 36 | Color_a, Dif_b, CWT, FIRM, SDS, GI, MIXO | I^†^ | 3.28 | 5 |
| 4A | 139.2-143.7 | 44 | Color_a, Color_b, Color | I^††^, II^††^ | 2.72 | 4.1 |
| 7A | 180.3-181.8 | 88 | Color_b, Dif_L, FIRM, CWT, WTS | I^†^, II^††^ | 3.22 | 5 |
| 7B | 132.9 | 92 |  | I^††^, II^††^ | 2.12 | 3 |
| 7B | 196.5 | 95 | Color_b | I^††^, II^††^ | 2.39 | 3.5 |
| **Polyphenol oxidase activity (PPO)** | |  |  |  |  |  |
| 1A | 6.6 | 1 | SDS, GI, FIRM,CWT, Color_a | I, II^†^ | 5.14 | 8.3 |
| 1B | 150.9-152 | 10 | VIT | I^††^, II^††^ | 2.61 | 3.9 |
| 2B | 120.2-124.9 | 21 | CWT | I^†^, II | 6.67 | 11 |
| 3A | 170.1-176.9 | 32 | GI, CWT | I, II | 9.03 | 15 |
| 3A | 183.8-184 | 33 |  | I, II | 7.49 | 12.3 |
| 3B | 190.4 | 38 |  | I, II | 9.03 | 15 |
| 3B | 198.5-205.1 | 39 |  | I, II | 9.03 | 15 |
| 5A | 136.3-141.4 | 54 | CWT | I^††^, II^††^ | 2.48 | 3.6 |
| 5A | 167.1-167.4 | 57 |  | I^††^, II^†^ | 3.39 | 5.2 |
| 5B | 63.4 | 59 |  | I^†^, II | 5.95 | 9.7 |
| 6A | 105.7 | 67 |  | I^†^, II^†^ | 3.84 | 6 |
| 6B | 27.1 | 72 |  | I^†^, II^†^ | 5.15 | 8.3 |
| 7B | 13.8-15 | 89 | Dif_L | I^††^, II^††^ | 2.77 | 4.2 |
| **e.        Cooking related traits** | | | | | | |
| **Firmness (FIRM)** | |  |  |  |  |  |
| 1A | 1.3-4.6 | 1 | SDS, GI, CWT, PPO, Color_a | III† | 3.27 | 5.1 |
| 1B | 3-8.5 | 4 | CWT, GI, MIXO, SDS | II††, III† | 4.93 | 8.1 |
| 3A | 7.3-9 | 27 | CWT | I††, II†† | 2.71 | 4.1 |
| 3B | 4.2-7.4 | 34 | CWT, CLOSS, WTS | I†, II† | 3.75 | 5.9 |
| 3B | 79.1-86.9 | 36 | CWT, SDS, GI, MIXO, TYP, Color_a, Dif_b | I††, II†† | 2.51 | 3.8 |
| 5A | 113.7-115.8 | 52 | CWT, WTS | III† | 4.1 | 6.6 |
| 6A | 113.5 | 68 | SPROT, WG | III† | 4.24 | 6.9 |
| 6B | 92.6 | 75 | CWT | III† | 3.63 | 5.8 |
| 7A | 180.3-184.1 | 88 | CWT, WTS, Dif_L, TYP, CWT | I††, II†† | 2.82 | 4.2 |
| 7B | 62.2-67.3 | 90 | WPROT, CWT, SDS | I††, III†† | 2.9 | 4.5 |
| **Cooking loss (CLOSS)** | |  |  |  |  |  |
| 3A | 21.3 | 28 | Color_a | I† | 4.21 | 6.7 |
| 3B | 4.2-7.4 | 34 | FIRM, CWT, WTS | I††, II† | 3.75 | 5.9 |
| 4A | 129.3 | 43 |  | I††, III†† | 2.45 | 3.6 |
| 4B | 17.7-22.5 | 46 | Dif_b, Color, Color_a, WG, VIT | III† | 3.27 | 5.1 |
| 4B | 105.5-106 | 48 | Dif_b | I | 3.85 | 6 |
| 6A | 67.9-72.4 | 64 | SDS, GI, GLUT | III† | 3.73 | 6 |
| 6A | 127.1-130.0 | 71 | Color_b | II††, III†† | 2.56 | 3.8 |
| **Work to shear (WTS)** | |  |  |  |  |  |
| 2B | 153.4 | 23 | CWT | I^††^, II^††^ | 2.32 | 3.4 |
| 3B | 4.2-7.4 | 34 | CWT, FIRM, CLOSS | I^†^ | 4.43 | 7 |
| 5A | 113.7-115.8 | 52 | FIRM, CWT | I^†^ | 3.2 | 4.9 |
| 7A | 180-184.1 | 88 | FIRM, CWT, Color_b, Dif_L, TYP | II^†^ | 3.02 | 4.6 |
| **Cooked weight (CWT)** | |  |  |  |  |  |
| 1A | 1.3-8.5 | 1 | SDS, GI, FIRM, PPO, Color_a | I^†^ | 4.33 | 6.8 |
| 1B | 3-8.5 | 4 | FIRM, GI, MIXO, SDS | I^†^, III^†^ | 3.8 | 6.2 |
| 1B | 15.2 | 5 | GI, SDS, MIXO | III^†^ | 4.7 | 7.7 |
| 1B | 27.6 | 6 |  | I | 6.11 | 10 |
| 1B | 50.3-54.8 | 7 |  | III^†^ | 3.47 | 5.5 |
| 2A | 143.2 | 11 | TEXT, SEXT | III | 4.9 | 8 |
| 2B | 44.5-44.7 | 18 |  | I^†^ | 3.88 | 9.1 |
| 2B | 53.4-56.4 | 19 |  | I^†^ | 3.4 | 5.3 |
| 2B | 80.6-84 | 20 |  | I^†^, III^†^ | 4.77 | 7.8 |
| 2B | 124.5-129.8 | 21 | PPO | I | 8.17 | 14 |
| 2B | 153.4 | 23 | WTS | I^††^, II^††^ | 2.3 | 3 |
| 3A | 7.3-9 | 27 | FIRM | I^†^, III^††^ | 3.85 | 6 |
| 3A | 170.1-176.9 | 32 | GI, PPO | I | 7.32 | 12 |
| 3B | 4.2-7.4 | 34 | FIRM, CLOSS, WTS | II, III^††^ | 4.3 | 7 |
| 3B | 75.5-86.9 | 36 | SDS, GI, FIRM, MIXO,TYP, Color_a, Dif_b | I | 8.14 | 13 |
| 5A | 113.7-115.8 | 52 | FIRM, WTS | I^††^ | 2.1 | 7.5 |
| 5A | 134.5-140.6 | 54 | PPO | I | 3.33 | 5.1 |
| 5A | 153.6-156.5 | 56 |  | I^††^, III^††^ | 2.61 | 3.9 |
| 6B | 92.6 | 75 | FIRM | I^†^ | 3.37 | 5.2 |
| 7A | 148.1 | 87 |  | I^†^ | 3.41 | 5.3 |
| 7A | 180.3-184.1 | 88 | FIRM, WTS, Dif_L, TYP, FIRM | III^†^ | 3.26 | 5.1 |
| 7B | 62.2-67.3 | 90 | WPROT, FIRM, SDS | I^††^, III^††^ | 2.47 | 3.6 |
| ^a^ cM, marker position on the consensus durum map of Maccaferri et al. (2015)  ^b^ I= Prosper balanced trial; II= Langdon balanced trial; III= unbalanced combined data set where an SNP marker was detected above the pFDR value  *the specific model to used identify significant MTAs for individual traits are reported in supplementary table 2  ^†^ SNP marker that was detected above –log_10_ (*P* value) of 3, but below the pFDR value in that trial (environment)  ^††^ SNP marker that was detected above –log_10_ (*P* value) of 2, but below the pFDR value in that trial (environment)  ^¶^R^2^, proportion of phenotypic variation explained by the individual marker  ## GLUT, WTS, Color_L, Color_a, color_b, Dif_L, Dif_a, Dif_b, PPO and TYP were only measure in balanced dataset, while TEXT, SEXT, SASH, MIXO and Color were only measured in historic dataset. | | | | | | |
